# Correction: CAPER Is Vital for Energy and Redox Homeostasis by Integrating Glucose-Induced Mitochondrial Functions via ERR-α-Gabpa and Stress-Induced Adaptive Responses via NF-κB-cMYC

**DOI:** 10.1371/journal.pgen.1005316

**Published:** 2015-06-15

**Authors:** The PLOS Genetics Staff

## Notice of Republication

This article was republished on May 26, 2015, to include details of the editor who handled the submission, which had been omitted due to an error in production. Please download this article again to view the correct version. The originally published, uncorrected article and the republished, corrected article are provided here for reference.

## Supporting Information

S1 File Originally published, uncorrected article. (PDF)

S2 File Republished corrected article. (PDF)
