# Supplementary figures and images for: CAPER Is Vital for Energy and Redox Homeostasis by Integrating Glucose-Induced Mitochondrial Functions via ERR-α-Gabpa and Stress-Induced Adaptive Responses via NF-κB-cMYC
Source: PLoS Genet. 2015 Apr 1;11(4):e1005116. doi: 10.1371/journal.pgen.1005116 (PMC4382186; doi:10.1371/journal.pgen.1005116)

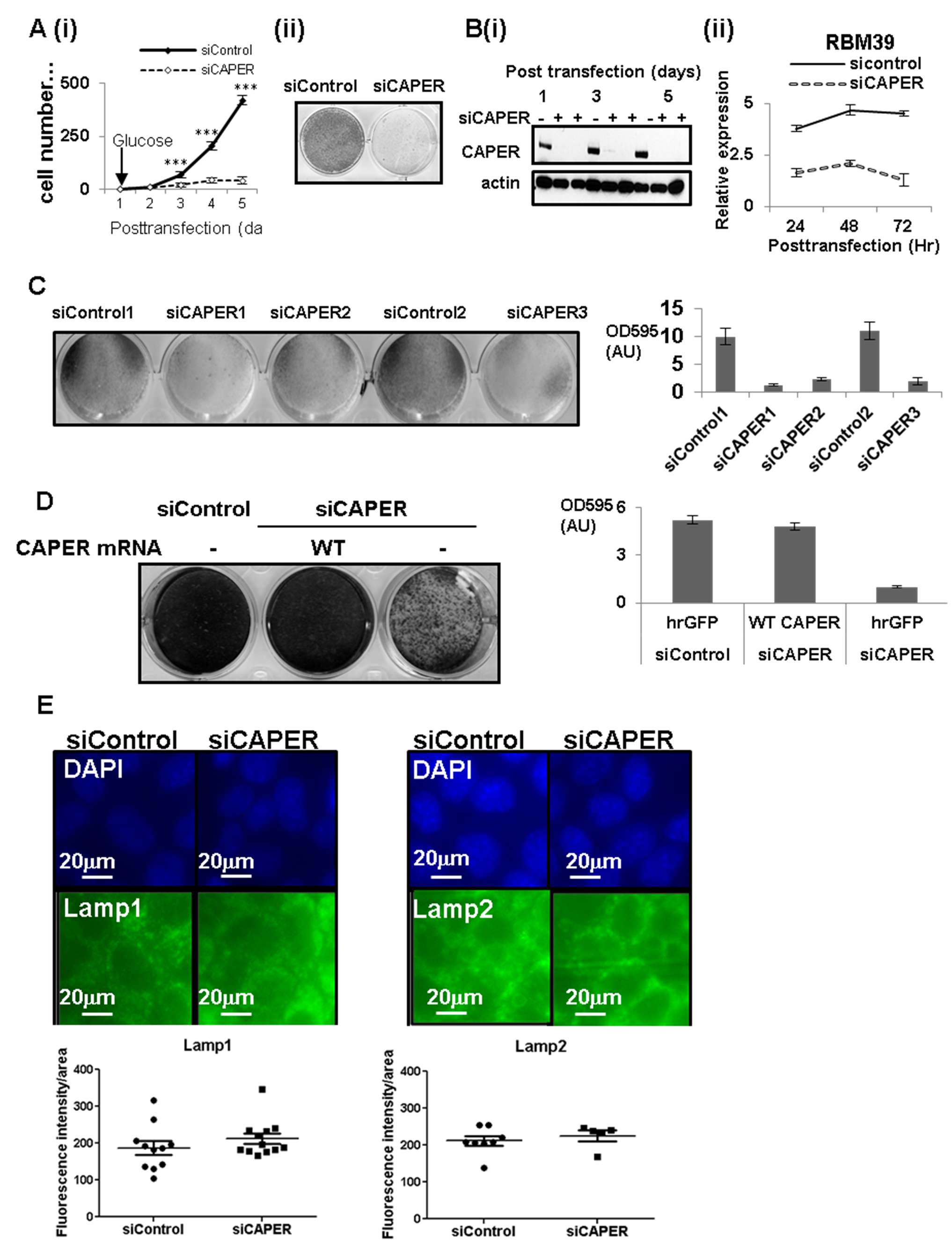

Supplement: S1 Fig — A. Knockdown of CAPER suppresses cell proliferation starting from 3 days. (i) A growth curve showing overall growth kinetics. Cell numbers were counted at the indicated days after transfecting either siControl (black solid line) or siCAPER (black dotted line). A graph presents average and SEM of the triplicate as an error bar. Statistical analysis by student’s t-test was performed (** p<0.01). (ii) A representative microscopic picture showing the result of crystal violet staining 5 days after transfection with indicated siRNAs. B. The efficacy and duration of siRNA targeting CAPER was confirmed by Western blots (i) and qRT-PCR analyses (ii). Amount of CAPER protein and mRNA were shown at the indicated days after transfecting each siRNA (-: control siRNA, +: siRNA targeting CAPER). C. A representative microscopic picture (left panel) and a quantitative graph (right panel) showing the result of crystal violet staining 6 days after transfection with indicated siRNAs targeting CAPER or control. Bar graphs present the mean value of three replicates and error bars represent SEM. D. A representative microscopic picture (left panel) and a quantitative graph (right panel) showing the result of crystal violet staining 6 days after transfection with indicated siRNAs targeting CAPER or control followed by transfection of either hrGFP or CAPER mRNAs. Bar graphs present the mean value of three replicates and error bars represent SEM. E. Fluorescence pictures (upper panel) and the corresponding quantitative graphs (lower panel) showing the relative abundance of mouse Lamp1(left panel) and Lamp2 (right panel)from the cells after transfection with either siControl (left panel) or siCAPER. Pictures were taken at 48 hours after siRNA transfection as described in the experimental procedure (right panel). A scale bar is present. Staining intensity was quantified by Image J software (NIH) and statistical significance was calculated by student’s t-test using GraphPad software. (TIF) [file pgen.1005116.s001.tif]

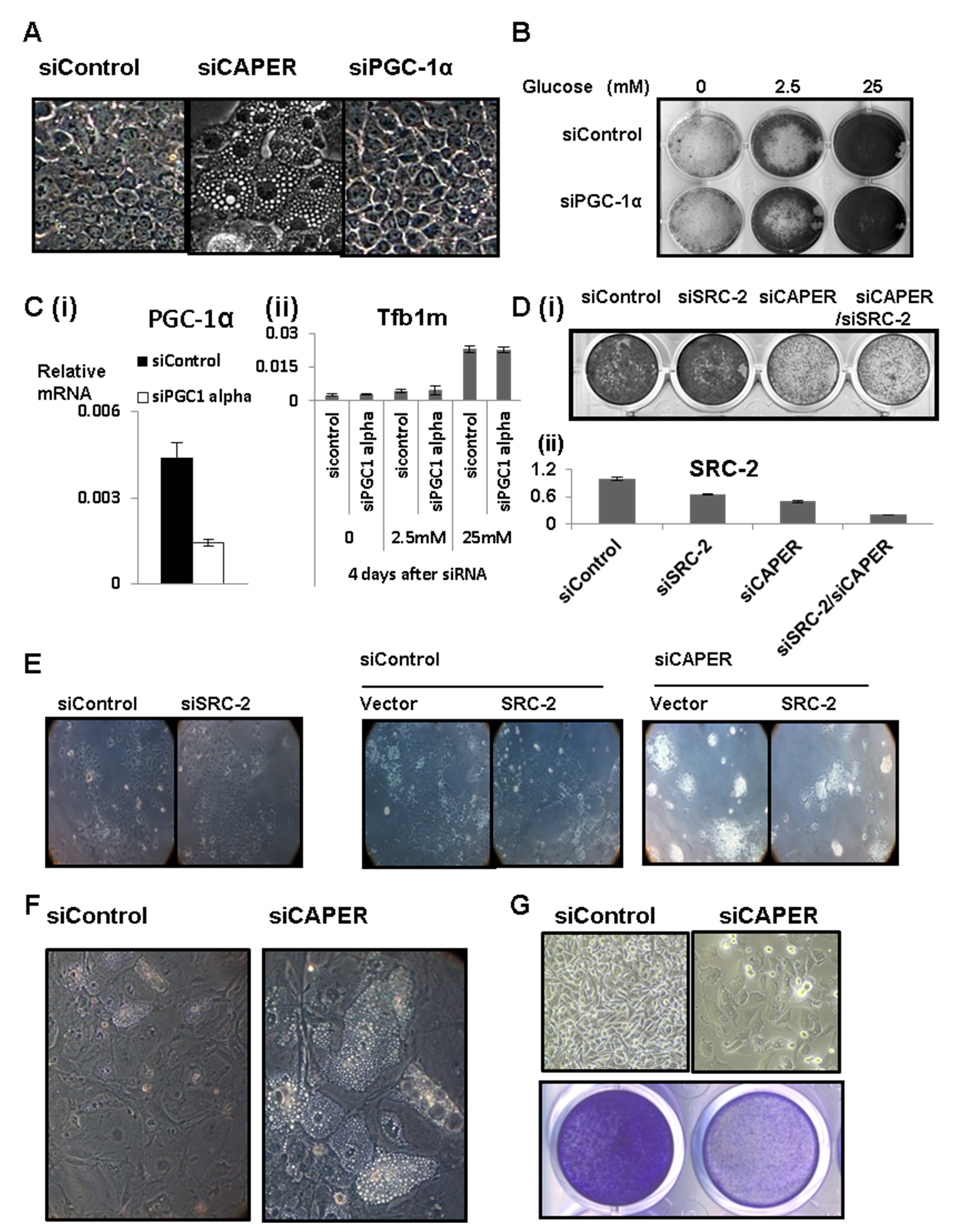

Supplement: S2 Fig — A. Microscopic pictures showing morphology of cells at 2 days after transfecting with siControl, siCAPER and siPGC-1α. B. Crystal violet staining showing the effect of siControl and siPGC-1α in combination with the indicated glucose concentration (mM) on cell proliferation. Photos were taken 6 days after siRNA transfection. C. A graph showing the result from qRT-PCR to measure mRNA levels of PGC-1α (i) and Tfb1m (ii) to confirm the efficacy of siPGC-1α(i) and its effect on glucose dependent induction of Tfb1m (ii), respectively. Levels of each transcript are normalized with the amount of β-2MG as an internal control. Each bar represents the mean value of normalized mRNA levels of each gene from triplicates with an error bar (SEM). Data were analyzed by t-test using GraphPad software. D. (i) Crystal violet staining showing the effect of siControl, siCAPER and siSRC2 on cell proliferation. Photos were taken 6 days after siRNA transfection. (ii) A graph showing the qRT-PCR result measuring mRNA of SRC2 after transfecting with the indicated siRNAs. Quantitation and statistical analyses are as described in the Materials and Methods. E. Microscopic pictures showing morphology of the cells transfected with either the indicated siRNAs only or in combination of Adenovirus encoding vector or SRC-2. 6 hours after siRNA, adenoviruses were infected with 8microgram/ml of polybrene in serum free media for overnight. Photos were taken 3 days after adenovirus infection. F. A representative microscopic picture shows the morphology of primary hepatocytes 2 days after transfection with indicated siRNAs targeting CAPER or control. G. Representative microscopic pictures show the morphology of Hepa1-6 cells 2 days after transfection (upper panel) and the result of crystal violet staining 6 days after transfection (lower panel) with indicated siRNAs targeting CAPER or control. (TIF) [file pgen.1005116.s002.tif]

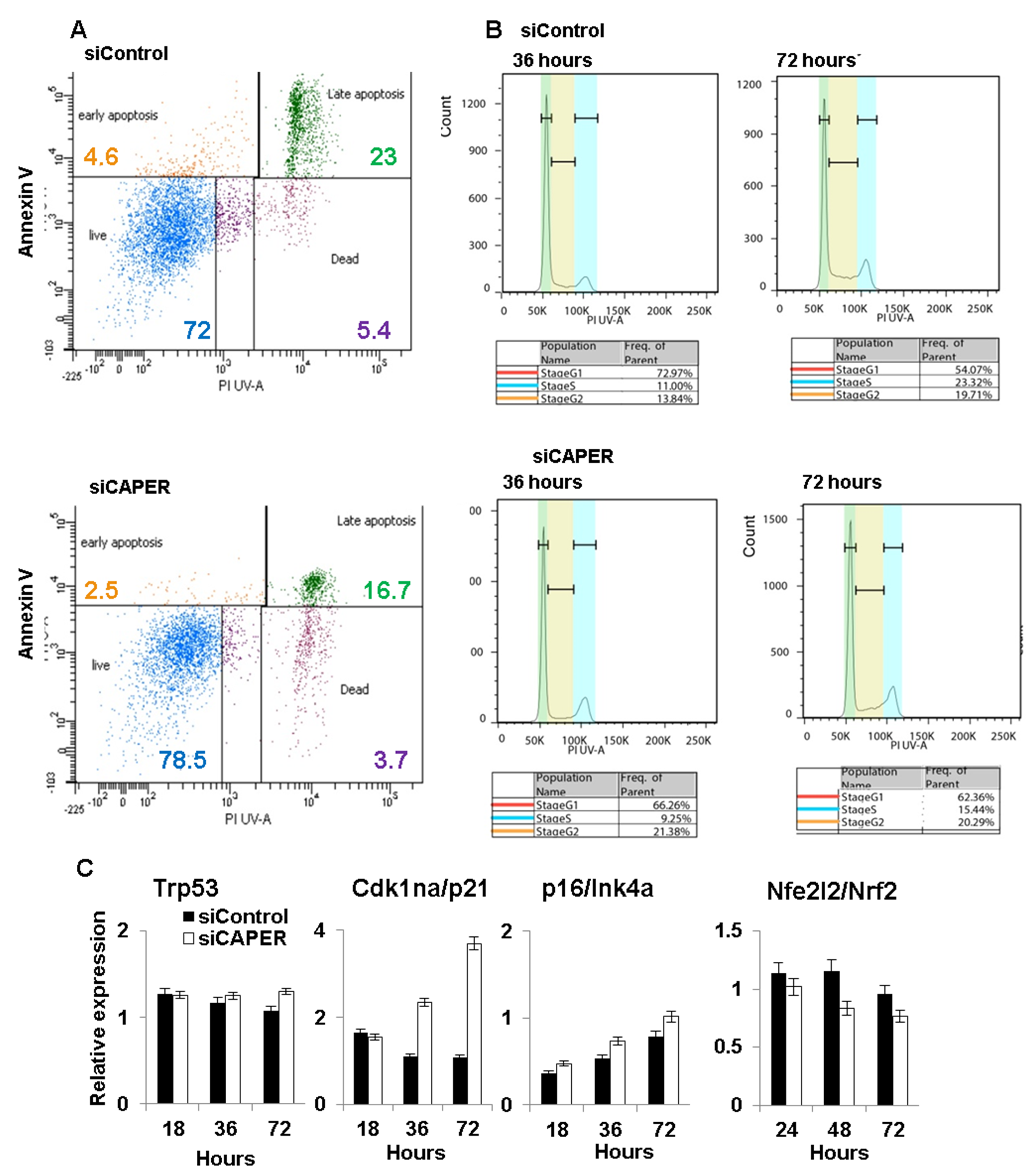

Supplement: S3 Fig — A. FACS analyses showing proportion of Annexin V positive cells from cells depleted CAPER (siCAPER) and control (siControl). Annexin V staining was performed 3 days after transfecting either control (upper panel) or siCAPER (lower panel). B. Histogram showing the FACS analyses of PI staining at 36 hours (left) and 72 hours (right) after transfecting either control (upper panel) or siCAPER (lower panel). C. QRT-PCR showing the kinetic expression of p53, p21, p16/Ink4 and Nfe2l2/Nrf2 transcripts at the indicated times after transfection. Quantitation and statistical analyses were carried out as described in S2C Fig. (TIF) [file pgen.1005116.s003.tif]

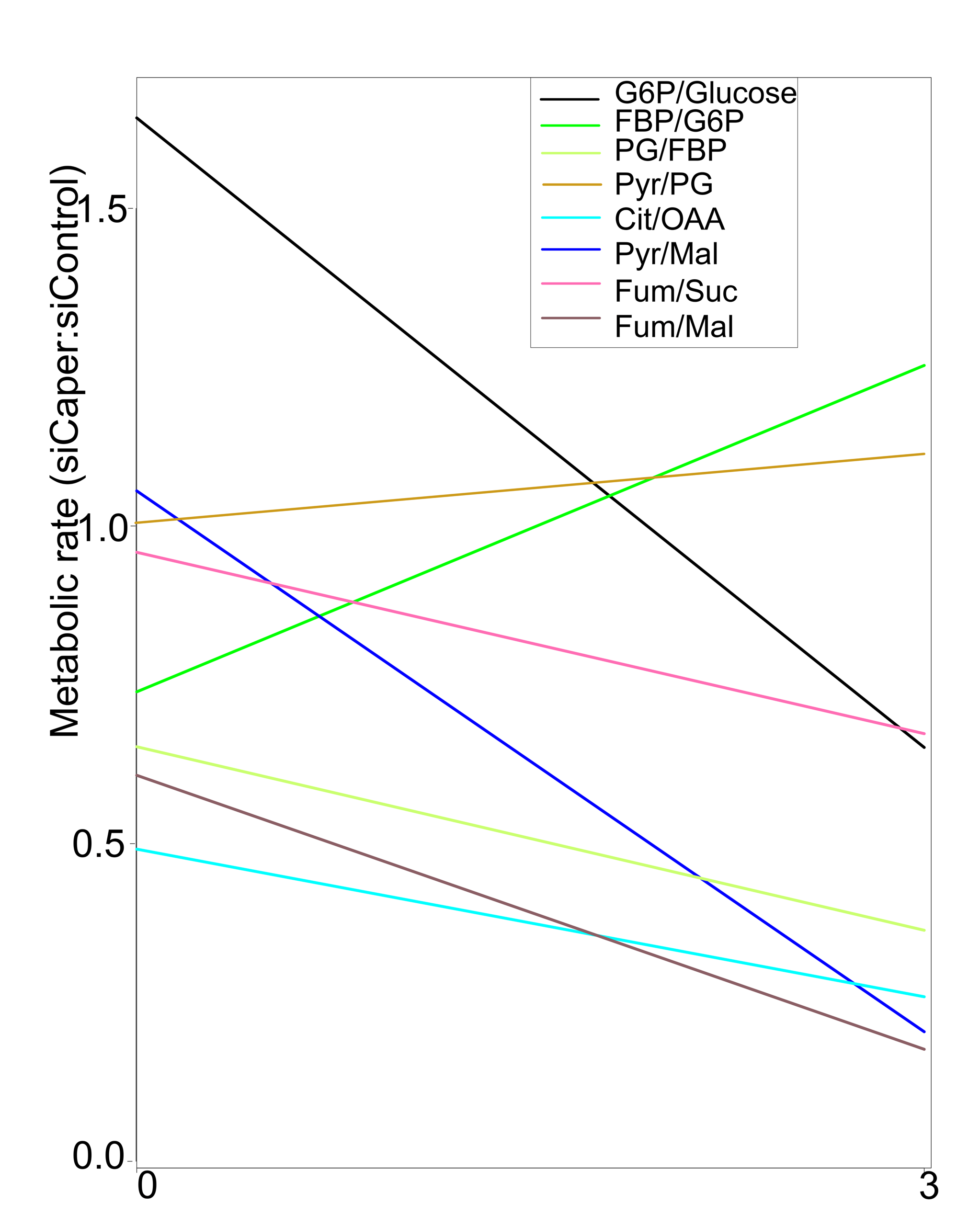

Supplement: S4 Fig — A graph showing the computed ratio of the rate of average total quantities of two C13 labeled metabolites in siCAPER to ones in siControl cells. We computed the ratio of two indicated metabolites from cells treated with siCAPER or siControl. We then calculate the mean value of these ratios of metabolites in three biological replicates. Using these mean values of metabolic rate, we computed the ratio of these values of cells treated with siCAPER to ones with siControl. Each replicate is a pool of biological duplicate. (TIF) [file pgen.1005116.s004.tif]

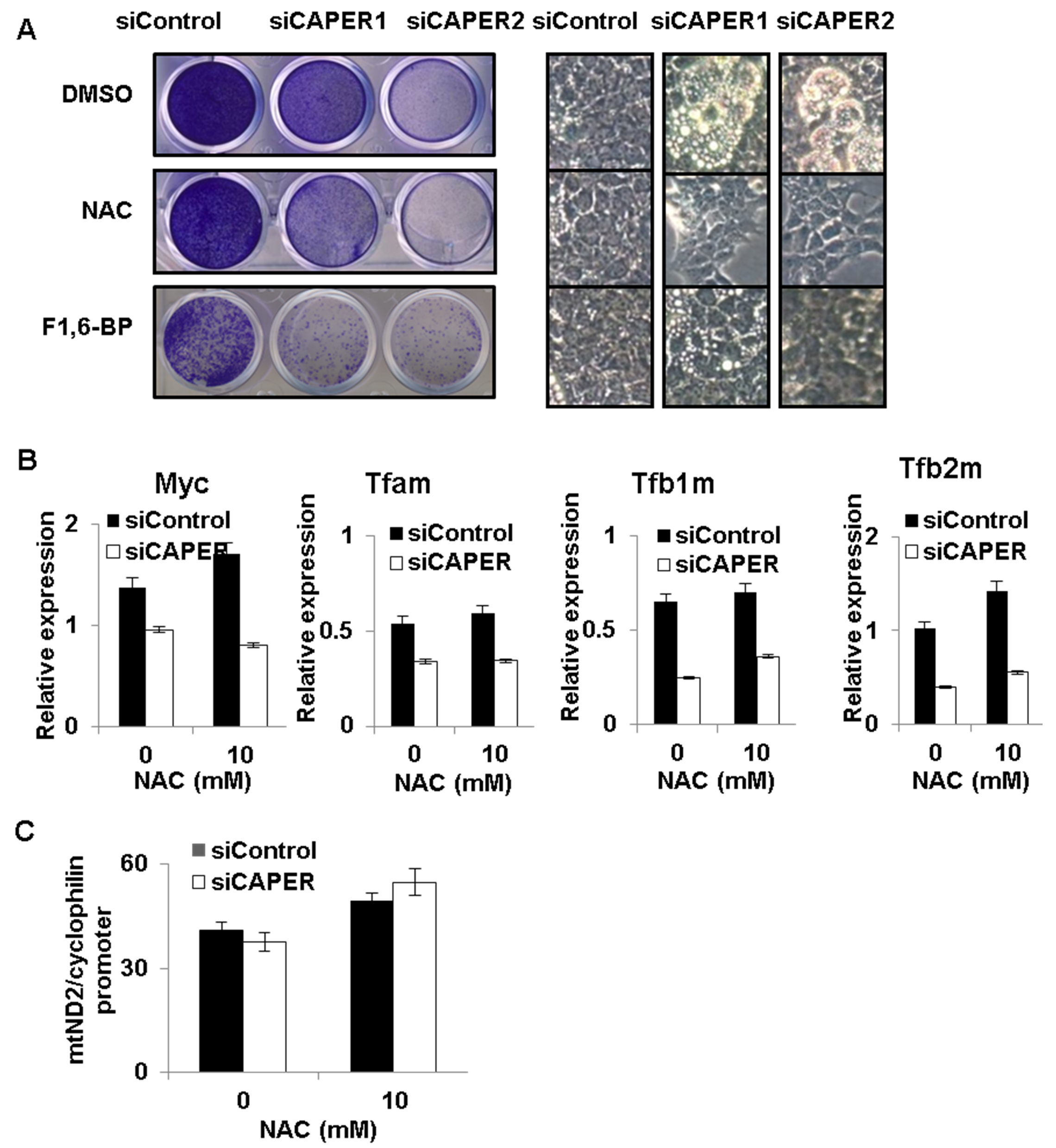

Supplement: S5 Fig — A. Microscopic pictures showing the result of crystal violet staining (left) and morphology (right) of the cells treated with two different siRNAs targeting CAPER (siCAPER1 and siCAPER 2) followed by treating NAC and F1,6-BP. B. Quantitative graphs of qRT-PCR showing the negligible effect of NAC treatment on the levels of c-Myc, Tfam, Tfb1m and Tfb2m from the cells treated with either control or siCAPER. C. Quantitative graphs of qRT-PCR showing the comparable ratio of mitochondrial DNA copy number to nuclear DNA copy number with or without NAC. (TIF) [file pgen.1005116.s005.tif]

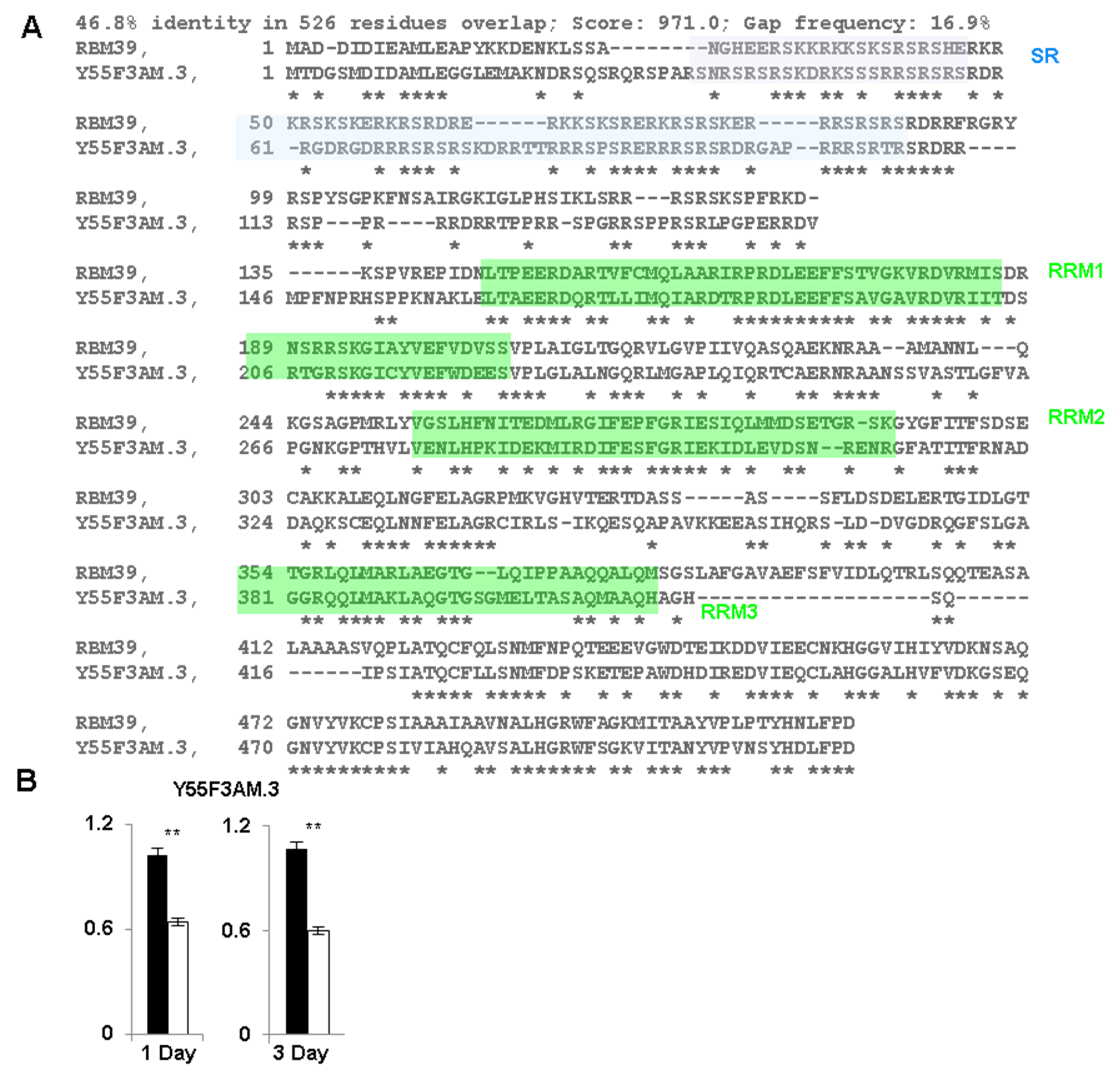

Supplement: S6 Fig — A. Amino acid sequence alignment of Y55F3AM.3 and hRBM39. B. A qRT-PCR showing the reduced full-length Y55F3AM.3 in both 1-day (left) and 3-day (right) old worms fed bacteria expressing either control (balck) or Y55F3AM.3 (white) RNAi clones. (TIF) [file pgen.1005116.s006.tif]

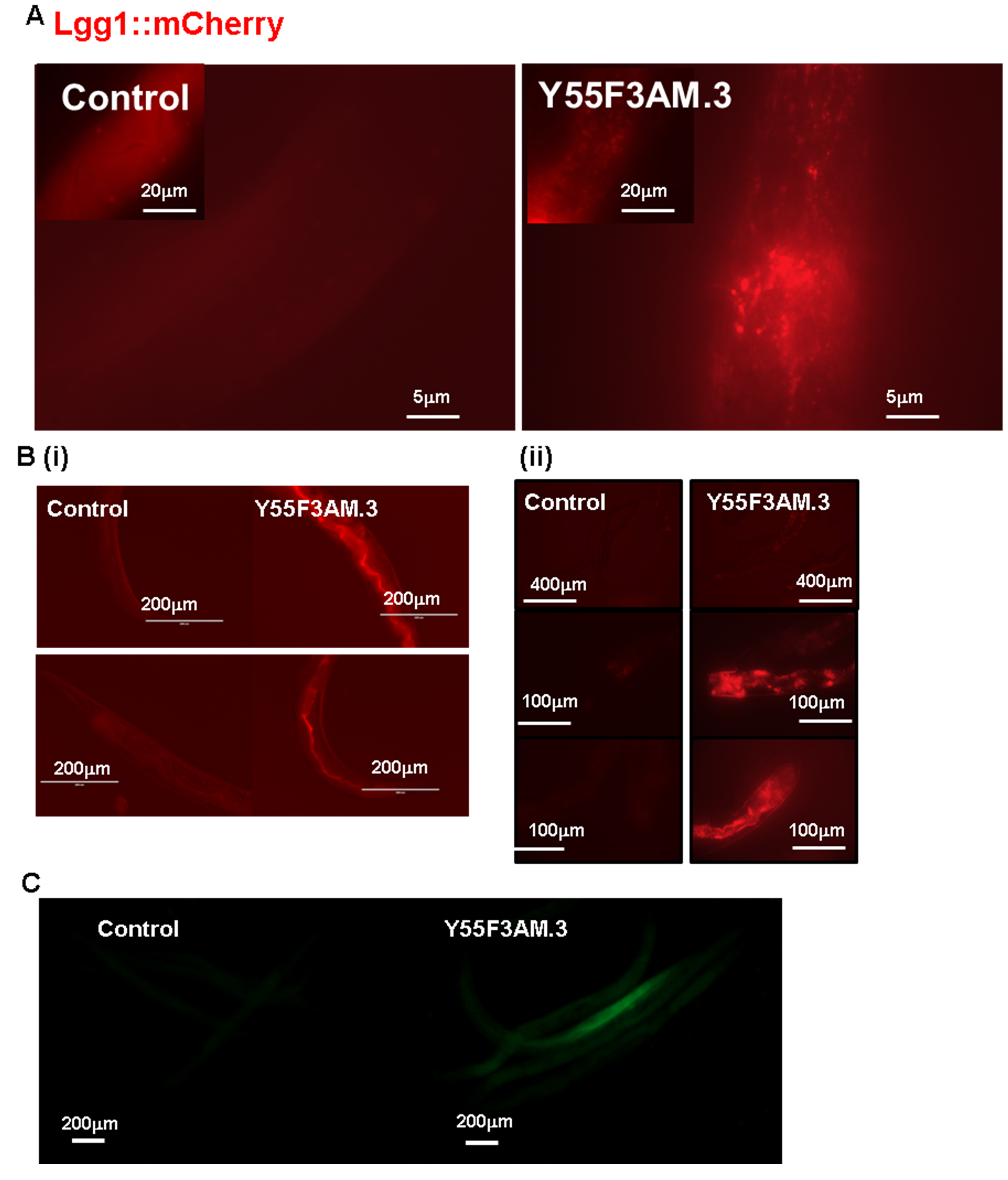

Supplement: S7 Fig — A. Representative microscopic pictures showing the signals of mCherry::Lgg-1 in 5-day old adult animals fed bacteria expressing either control or Y55F3AM.3 RNAi clones since L1 stage. B. Representative microscopic pictures show the lysotracker staining of either control or Y55F3AM.3-inactivated 3 (i) or 5-day (ii) old adult rrf-3 (pk1426) strains fed with the indicated RNAi clones since L1 stage. C. Representative microscopic pictures show the lysosensor DND-189 staining of either control or Y55F3AM.3-inactivated 5-day old adult rrf-3 (pk1426) strains fed with the indicated RNAi clones since L1 stage. (TIF) [file pgen.1005116.s007.tif]
