# Supplementary material for: CAPER Is Vital for Energy and Redox Homeostasis by Integrating Glucose-Induced Mitochondrial Functions via ERR-α-Gabpa and Stress-Induced Adaptive Responses via NF-κB-cMYC
Source: PLoS Genet. 2015 Apr 1;11(4):e1005116. doi: 10.1371/journal.pgen.1005116 (PMC4382186; doi:10.1371/journal.pgen.1005116)
Supplement: S1 Table — (DOCX) [file pgen.1005116.s008.docx]

**Table S1. Pairwise Alignment scores to compare human RBM39 gene with orthologs from 20 representative species.**

|  | Gene | Identity (%) | | Comparison |
| --- | --- | --- | --- | --- |
| Species | Symbol | Protein | DNA |  |
| H.Sapiens (human) | RBM39 |  |  |  |
| vs. P.troglodytes (Chimpanzee) | RBM39 | 100.0 | 99.8 | Blast |
| vs. C.lupus (Wolf) | RBM39 | 100.0 | 95.7 | Blast |
| vs. B.taurus (Cow) | RBM39 | 100.0 | 95.5 | Blast |
| **vs. M.musculus (Mouse)** | **Rbm39** | **99.8** | **93.1** | **Blast** |
| vs. R.norvegicus (Rat) | Rbm39 | 99.8 | 93.3 | Blast |
| vs. G.gallus (Chicken) | RBM39 | 98.5 | 86.0 | Blast |
| vs. A. Carolinensis (lizard) | RBM39 | 98 |  |  |
| vs. X. Laevis (African frog) | XI.4395 | 83 |  |  |
| vs. D.rerio (Zebra fish) | rbm39a | 82.8 | 72.0 | Blast |
| vs.D.melanogaster (Fruit fly) | CG11266 | 60.2 | 55.4 | Blast |
| vs. A.gambiae (Mosquito) | AgaP | 58.2 | 52.0 | Blast |
| **vs. C.elegans (Worm)** | **Y55F3AM.3** | **51.1** | **51.0** | **Blast** |
| vs. S.pombe (Fission yeast) | rsd1 | 38.6 | 44.9 | Blast |
| vs. M.oryzae (Fungus) | MGG_12101 | 43.0 | 46.8 | Blast |
| vs. N.crassa (Bread mold) | NCU03491 | 41.7 | 45.7 | Blast |
| vs. A.thaliana (Thale cress) | AT2G16940 | 43.1 | 48.2 | Blast |
| vs. O.sativa (Rice) | Os10g0439600 | 40.5 | 47.9 | Blast |
| vs. S. cerevisiae (Baker’s yeast) | RNA15 | 18 | 10 | Blast |
| vs. S. cerevisiae (Baker’s yeast) | NOP13 | 19 | 14 | Blast |
