# Supplementary material for: CAPER Is Vital for Energy and Redox Homeostasis by Integrating Glucose-Induced Mitochondrial Functions via ERR-α-Gabpa and Stress-Induced Adaptive Responses via NF-κB-cMYC
Source: PLoS Genet. 2015 Apr 1;11(4):e1005116. doi: 10.1371/journal.pgen.1005116 (PMC4382186; doi:10.1371/journal.pgen.1005116)
Supplement: S2 Table — (DOCX) [file pgen.1005116.s009.docx]

**Table S2. eQTL located in human RBM39 gene .**

| **RGD ID** | Name | LOD | P Value | Trait | Sub Trait | Species | Type |
| --- | --- | --- | --- | --- | --- | --- | --- |
| **2314820** | Glucose level QTL 124 (human) | 2.27 | 0.0002 | Glucose level | fasting | Human | qtl |
| **1643578** | Glucose level QTL 32(human) |  | 0.003 | Glucose level | non-insulin-dependent | Human | qtl |
| **2316034** | Glucose level QTL 220 (human) |  | 0.00102 | Glucose level |  | Human | qtl |
| **2314562** | Glucose level QTL 57 (human) | 1.1 |  | Glucose level |  | Human | qtl |
| **2316664** | Glucose level QTL 249 (human) | 2.63 |  | Glucose level |  | Human | qtl |
| **2316720** | Glucose level QTL 288 (human) | 1.72 | 0.00432 | Glucose level |  | Human | qtl |
