# Supplementary material for: CAPER Is Vital for Energy and Redox Homeostasis by Integrating Glucose-Induced Mitochondrial Functions via ERR-α-Gabpa and Stress-Induced Adaptive Responses via NF-κB-cMYC
Source: PLoS Genet. 2015 Apr 1;11(4):e1005116. doi: 10.1371/journal.pgen.1005116 (PMC4382186; doi:10.1371/journal.pgen.1005116)
Supplement: S1 Text — (DOCX) [file pgen.1005116.s014.docx]

**Supplementary Information**

**Extended Methods**

**Reagents, Cell Culture, Transient transfection and Determination of Cell proliferation.**

D-glucose, D-Fructose 1, 6-bisphosphate trisodium salt hydrate (F1,6-BP), Chloroquine, E64d, TMRM (Tetramethylrhodamine, methyl ester), FCCP(Carbonyl cyanide 4-(trifluoromethoxy phenylhydrazone), Rotenone, Antimycin A and Oligomycin were purchased from Sigma. DAPI (4′,6-diamidino-2-phenylindole, dihydrochloride), Mitotracker Red CMX Ros, Lysosensor Green DND-189 and Lysotracker Red DND-99were purchased from Invitrogen (Molecular Probe). AML12 cells were purchased from ATCC and cultured in 1: 1 mixtures of Dulbecco's modified Eagle's medium and Ham’s F12 medium (DMEM/F12) with 10% v/v FCS supplemented with 0.005 mg/ml insulin, 0.005 mg/ml transferrin, 5 ng/ml selenium, and 40 ng/ml dexamethasone . All siRNA oligos were purchased from Thermo Scientific. Individual siRNAs derived from SMRT pool were used to knock down CAPER in cells. Cells were transfected with 30 nM each siRNA with lipofectamine 2000 according to the manufacturer’s instruction (Invitrogen) then harvested. Half of the cells were subjected to Western blot analysis and the other half of the cells were subjected to qRT-PCR. Luciferase assays were carried out as previously reported [1]. Transfection of in vitro transcribed mRNAs was performed using calcium phosphate (Invitrogen) following the manufacturer's instructions. To overexpress CAPER, we infected adenovirus encoding either vector alone or full-length CAPER under CMV promoter at MOI 100 in the media containing 8 μl/ml of polybrene for 24 hours. 3 and 6 days after infection, pictures were taken at 10X. Crystal violet assay was done to score cell proliferation.

To determine cell proliferation, an equal numbers of cells were seeded in 12-well plates. Cell proliferation was determined every other day by counting cell numbers. For crystal violet staining, 24,000 cells of each set of triplicates were seeded in 12-well plates and were grown in the absence or presence of siRNA treatment. After 6 days of growth, cells were stained with crystal violet (Sigma) for 15 min at room temperature. For quantitation, stained crystal violet was extracted with 10% acetic acid and the intensity of color was measured by photospectormetry at OD595.

For cell cycle analyses, PI staining with FACS analyses were performed per manufacturer’s instruction using Propidium Iodide Nucleic Acid Stain (Molecular probes). To examine apoptosis, Annexin V staining was carried out per manufacturer’s instruction using Annexin V staining kit (Molecular Probes). 10000 cells were scored by LSR II. PI staining was analyzed by Flow Jo software and Annexin V staining was analyzed by DiVa.

**ATP measurement**

Cellular ATP was measured by ATP lite kit (Perkin Elmer) according to manufacturer’s instruction. After transfecting siRNA, 2000 cells were seeded and grown onto 96 well plates. After 24, 48 and 72 hours, cells were lysed with lysis buffer and we measured cellular ATP levels using luminometer.

### Immunocytochemistry, Staining, and Microscopy

For immunocytochemistry, transfected AML12 cells on the cover glass were fixed with 3.7% formaldehyde for 15 min at room temperature followed by permeabilization with 1X PBS with 0.1% of Triton X-100 and washing with 1X PBX three times. Cells were then incubated with 1X PBS containing 5% Nonspecific Goat Serum for 1 hour at RT. Monoclonal antibodies against LAMP-1 (1D4B) and LAMP-2 (ABL-93), developed by J. Thomas August, was obtained from Developmental Studies Hybridoma Bank, created by NICHD of the NI and maintained at The University of Iowa, Department of Biology, Iowa City, IA 52242. Cells on the cover glass were incubated with monoclonal antibodies at 1:250 in 1X PBS containing 5% Nonspecific Goat Serum at 4C for overnight followed by washing with 1X PBS three times. Cells were then incubated with secondary antibody conjugated with Alexa Fluoro488 (Invitrogen) at 1:500 for 1 hour. After washing with 1X PBX three times and DAPI staining, cells were fixed again with 3.7% formaldehyde for 15 min at room temperature. After washing with 1X PBS, cells were mounted with slow fade and subjected to microscopy analyses.

### For staining, transfected AML12 cells on the cover glass were treated with 500nM of Mitotracker red CMX Ros for 30minor 1 μM of Lysosensor green DND-189 for 5 minor 1 μM of Lysotracker red DND-99 for 10 min or 25μM of Carboxy-H2DCFDA for 30min, respectively at 37^o^C. Cells were then washed with 1X PBS three times and fixed with 3.7 % formaldehyde for 15 min at room temperature. Cells were then incubated with 1 μM DAPI followed by washing with 1X PBS three times followed by fixation with 3.7 % formaldehyde for 15 min at room temperature. The cover glasses were mounted onto the glass slide with Slow fade (Invitrogen) and subjected to microscope. Deconvolution microscopy was performed with a Zeiss Axiovert S100 TV microscope and a DeltaVision Restoration Microscopy System (Applied Precision, Inc.). Z series focal planes were digitally imaged and deconvolved with the DeltaVision constrained iterative algorithm. All image files were digitally processed for presentation with Adobe Photoshop.

**Mitochondrial analyses**

Mitochondrial numbers were measured by both using qPCR to measure the ratio between mtDNA (mtND2) and nuclear DNA (cyclophilin) and staining with Mitotracker Red CMX Ros (Molecular Probe). Briefly, AML12 cells transfected with siRNAs were seeded onto the cover glass. 36 hours after transfection, cells were stained with 200nM of Mitotracker Red CMX Ros for 30 min at 37^o^C followed by extensive washing with 1X PBS. Cells were then fixed with 3.7% formaldehyde for 15 min at RT. After washing with 1X PBS, cells were stained with DAPI for 5 min at RT. After mounting with Slow Fade, cells were subjected to the microscopic analyses. The mitochondrial oxygen consumption rate (OCR) and extracellular acidification rate (ECAR) were examined by XF24 analyzer (Seahorse bioscience) according to the manufacturer’s instructions. Briefly, cells were transfected with siRNAs and 3000 cells were seeded onto XF24 plates. After 24 hours, cells were subjected to the XF24 analyzer upon instructions.

**Reverse transcription and Real-Time Quantitative PCR**

QRT-PCR analyses of endogenous mRNAs were performed. Total RNA was isolated from 12-well culture dishes using the RNeasy kit (QIAGEN) followed by first strand cDNA synthesis using Superscript II (Invitrogen) according to the manufacturer’s instruction. Real-time PCR reactions were performed using the AIB Step One Plus sequence detection system (Applied Biosystems) with SyBr Green reaction mixture according to the manufacturer’s instruction. Means and S.E.M from the three or five independent samples were then calculated. The Student's t test was used to evaluate the data. To avoid variations from different samples, the relative mRNA level of each gene was normalized against the beta-2-microgloblulin mRNA content of the same sample. Primer sequences are available upon request.

**Western blot analyses and Quantitation**

Whole cell lysates were prepared as described previously[1]. To maintain the integrity and phosphorylation status of proteins, proteinase inhibitors and phosphatase inhibitors (Gene Depot) were added freshly. Western blot analyses were performed following standard procedures using Immune star (Bio Rad). Antibodies were from various sources: Anti-tubulin (Sigma), anti-NF-κB and anti-CAPER antibody (Bethyl) antibody is from Novus. Each band was subjected to quantitation as follows: relative fold-changes were presented by setting up each normalized protein to “1”- relative to the amount shown in lane 1 unless stated otherwise. Quantification of the relative intensity from the image was done using Image J free software (NIH, USA).

**Immunoprecipitation and Chromatin immunoprecipitation**

Immunoprecipitation and GST pull down assays were essentially performed as previously described [1]. To avoid masking signals by IgG, we used True blot IP beads (eBioscience). ChIP assay was performed using the Simple ChIP enzymatic Chromatin IP kit (Cell Signaling) according to manufacturer’s instructions. The Epitect ChIP qPCR primers for NF-κB binding sites for c-Myc and Frap1 were purchased from Qiagen.

**Metabolomic profiling**

Reagents and internal standards: High-performance liquid chromatography (HPLC) grade acetonitrile, methanol and water were purchased from Burdick & Jackson (Morristown, NJ). Mass spectrometry grade formic acid and internal standards namely, ([13C]2 Pyruvate, Zeatine, [15N]-Tryptophan, [D4] Thymine, [D27] Myristic acid and [15N] Arginine [15N] Anthranilic acid and [13C]4 Fumarate were purchased from Sigma- Aldrich (St.Louis, MO) or Cambridge Isotope Laboratories, Inc (Tewksbury, MA). The calibration solution containing multiple calibrants in acetonitrile/trifluroacetic acid /water was purchased from (Agilent Technologies, Santa Clara, CA).

The metabolomic analyses of all samples were executed using the protocol as described previously [2]. The raw peak intensity data (LC-MS output) was normalized using internal standards. Prior to normalization, the median coefficient of variation (CV) of each internal standard was measured to select the appropriate standard for normalization in order to confirm consistency.

A number of internal standards, including injection standards, process standards, and alignment standards were used to assure QA/QC targets were met and to control for experimental variability. Aliquots (200 µL) of 10 mM solutions of isotopically labeled standards were mixed and diluted up to 8000 µL (Final concentration 0.25 mM) and aliquoted into a final volume of 20 µL. The aliquots were dried and stored at -80 ^o^C until used further. The reproducibility of the profiling process was addressed at two levels; one by measuring only instrument variation, and the other by measuring overall process variation. To monitor instrument performance, 20 µL of a matrix-free mixture of the internal standards described above, reconstituted in 100 µL of methanol: water (50:50) was analyzed by MRM. In addition the process of metabolite extraction from the cell lines were monitored using pooled liver samples and spiked internal standards. In case of the former, the 100 mg of pooled liver was extracted in tandem with the cell lines using buffer containing spiked internal standards as described above. In addition, internal standards were also spiked into cell lines during the extraction process. Importantly, the matrix-free internal standards and liver samples were analyzed twice daily. The median coefficient of variation (CV) value for the internal standard compounds was 3.9%. To address overall process variability, metabolomic studies were augmented to include a set of nine experimental sample technical replicates (also called matrix, abbreviated as MTRX), which were spaced evenly among the injections for each day. Reproducibility analysis for the n=33 compounds were measured in each of these three replicate samples gave a measure of the combined variation for all process components including extraction, recovery, derivitization, injection, and instrument steps. The median CV value for the experimental sample for technical replicates (tissue profiling part of this study) was 14.6%.

Sample preparation for mass spectrometry-based examination of metabolome in Cell lines: Using 10 replicates per each condition, we measured the relative levels of metabolites in cells treated with siControl or siCAPER at 24 hours, Cell pellets were stored at -80 °C until analysis and cell pellets were thawed at 4 ^o^C and subjected to freeze-thaw cycle in liquid nitrogen and over the ice three times to rupture the cell membrane. For extraction of metabolome, 5 million of each group of cells (ten replicates) were homogenized in 1:4 ice cold water: methanol mixture containing an equimolar mixture of 8 standard compounds ([13C]2 Pyruvate, Zeatine, [15N]-Tryptophan, [D4] Thymine, [D27] Myristic acid and [15N] Arginine [15N] Anthranilic acid and [13C]4 Fumarate. This was followed by sequential addition of ice cold chloroform and water in 3:1 ratio and separation of the organic (methanol and chloroform) and aqueous solvents (water:methanol:chloroform:water; ratio 1:4:3:1). The aqueous extract was de-proteinized using a 3 KDa molecular filter (Amicon Ultracel -3K Membrane, Millipore Corporation, Billerica, MA) and the filtrate containing metabolites was dried under vacuum (Genevac EZ-2plus, Gardiner, NY). Prior to mass spectrometry, the dried extract was resuspended in an identical volume of injection solvent composed with appropriate mobile phase and subjected to liquid chromatography (LC) mass spectrometry.

Liquid Chromatography/Mass Spectrometry (LC/MS): The chromatographic separation of metabolites was performed using either reverse phase (RP) separation or Luna Amino (Phenominex) online with 6490 QQQ mass spectrometers (Agilent Technologies, Santa Clara, CA). Targeted profiling (SRM) for the Amino acids, the RP chromatographic method employed a gradient containing water (solvent A) and acetonitrile (ACN, solvent B, with both solvents containing 0.1% formic acid). Separation of metabolites was performed on a Zorbax Eclipse XDB-C18 column (50 × 4.6 mm i.d.; 1.8 μm, Agilent Technologies, CA) maintained at 37 °C. The binary pump flow rate was 0.2 ml/min with a gradient spanning 2% B to 95% B over a 25 minute time period. Targeting the TCA metabolites, the normal phase (NP) chromatographic separation was also used for targeted identification of metabolites. This employed solvents containing acetonitrile (ACN, solvent B): water (solvent A), with modified by the addition of 5mM Ammonium acetate (pH 9.9). The binary pump flow rate was 0.4 ml/min with a gradient spanning 80 % B to 2 % B over a 20 minute period followed by 2 % B to 80 % B for a 5 min period and followed by 80% B for 8 minute time period. Metabolites were separated on a Luna Amino column (4μm, 100A 2.1x150mm, Phenominex), that was maintained in a temperature controlled chamber (37 ^o^C). All the columns used in this study were washed and reconditioned after every 50 injections.

The mass spectrometry portion of the targeted profiling platform is based on a 1290 SL Rapid resolution LC and a 6490 triple Quadrupole (QQQ) mass spectrometer (Agilent Technologies, Santa Clara, CA). The samples were independently examined in both positive and negative ionization modes using a iFunnel Electrospray Ionization (ESI) source. The data acquisition during the analysis was controlled using the Mass Hunter workstation data acquisition software. To check the correlation with cell line and worm samples, the worm samples were analyzed on another 1290 SL Rapid resolution LC and a 6490 triple Quadrupole (QQQ) mass spectrometer (Agilent Technologies, Santa Clara, CA)

Isotope labeling and profiling by high resolution mass spectrometry: Glucose-free DMEM media was supplemented with 10% dialyzed serum and 4g/L 12C-glucose or U-13C6-glucose, (Cambridge Isotope Labs). For glucose-flux analysis, cells were maintained in glucose free DMEM media overnight. The next day the media was replaced with U-13C6-glucose containing media.

For metabolite collection, media from biological triplicates (in 10 cm dishes at 70% confluence) was fully aspirated; the cells were frozen by adding the liquid nitrogen. Metabolic extractions 1 ml of 50% (v/v) methanol was added. Cells and the metabolite-containing samples were collected into extraction vials. The cells were sonicated for 1 min (30 sec pulse twice) and mixed with 450 μl of ice cold chloroform and vortex mixed in a Multi-Tube Vortexer for 10 min. The resulting homogenate was mixed with 150 μl of ice cold water and vortexed again for 2 min. The homogenate was incubated at -20 ^o^C for 20 min and centrifuged at 4 ^o^C for 10 min to partition the aqueous and organic layers. The aqueous and organic layers were combined and dried at 37^o^C for 45 min in an Automatic Environmental Speed Vac® system (Thermo Fisher Scientific, Rockford, IL). The extract was reconstituted in 500 μl of ice cold methanol: water (50:50) and filtered through 3 KDa molecular filter (Amicon Ultracel -3K Membrane, Millipore Corporation, Billerica, MA) at 4 ^o^C for 90 min to remove proteins. The filtrate was dried at 37 oC for 45 min in speed vac and stored at -80 ^o^C until mass spectrometry analysis. Prior to mass spectrometry analysis, the dried extract was resuspended in 50 μL of methanol:water (50:50) containing 0.1% formic acid and analyzed using 6538 UHD Quadrupole Time of Flight (QTOF) accurate high resolution mass spectrometer. 5 μL of each extract was injected and analyzed using a 6538 QTOF mass spectrometer (Agilent technologies) coupled to a 1260 series HPLC system. The samples were examined in negative ionization modes using a dual Electrospray Ionization (ESI) source. Real time mass correction during mass spectrometry was achieved by infusion of a standard mixture of reference ions using an independent 1200 SL Rapid resolution LC isocratic pump equipped with 100:1 splitter to output a flow rate of 5 ml/min. This reference mixture supplied by the vendor contained ions with m/z 119.03632, 966.000725 for mass correction in the negative (-) ionization modes respectively. A mass range between 50-1000 m/z was employed for the entire process. The data acquisition during the analysis was controlled using the Mass Hunter workstation data acquisition software. For the metabolic flux experiments, mass spectral data was acquired in centroid modes. Samples were delivered to the MS via normal phase chromatography using a Luna Amino column (4um, 100A 2.1x150mm, Phenominex) at 400 ml/min. gradient spanning 80 % B to 2 % B over a 20 minute period followed by 2 % B to 80 % B for a 5 min period and followed by 80% B for 8 minute time period re-equilibrate the column. Buffer A was comprised of 5 mM ammonium acetate (pH = 9.9) in water and buffer B was 100% acetonitrile. To assess the validity of our method for calculating isotopomers, we determined the complete isotopomer distributions for each metabolite. Data analysis was performed in Quantitative analysis and estimated the % of isotopomer incorporation using the formula [% of Incorporation = (^13^C/ (^13^C+^12^C)) X100] after subtracting the natural abundance from the raw values.

We used only 13C6 [M+6] 13C incorporation to study forward flux in glycolysis up to the point where C3 intermediates are formed. In case gluconeogenesis we used 13C3 [M+3], TCA intermediates Pyruvate, PG and PEP we used 13C2 [M+2] 13C3 [M+3] 13C4 [M+4] and Malate & Citrate we used the 13C2 [M+2] 13C3 [M+3] to study metabolic flux [3,4]

#### Statistical Analysis of Metabolomics: The raw peak intensity (LC-MS output) was normalized using internal standards, for three different groups of metabolites. Prior to normalization, the CV of each internal standard was measured to select the appropriate standard for normalization. Accompanying box-plot of additional liver samples which were run along the experimental samples were also used to confirm instrumental consistency. The TCA dataset was normalized using L-Zeatine, while a dataset containing amino acids and fatty acids were normalized using labeled Arginine and Tryptophan respectively. The normalized data was log-transformed (log2) and used for t-test to identify differential metabolites between control and siCAPER groups based on p-values. A group of significant metabolites were identified based on Fold Discovery Rate (FDR) adjusted p-value (p.adjust() function in R) to mitigate the effects of multiple hypotheses testing. The median based fold change calculation was also performed on all three datasets and their respective values were documented. The heatmap presented here is based on the Spearman’s correlation criteria using the log transformed data. All the data analyses were performed using R^®^ [5] statistical software and dependent packages (DEDS [6] Sparcl [7], gplots [8].

The % of total C13 incorporation into the metabolites were calculated for each metabolites at every time points after taking the average of total C13 (normalized for natural abundance in each positional isomer) from the biological triplicates in each group (siCAPER and siControl) and plotted with SE and significance level (after comparing them in two sided t-test) at each time points (5min, 1, and 3 hours) using psych [9]. In addition, the ratio of total C13 relative quantification was measured after taking the average of the three biological replicates in both groups and plotted for 5min and 3 hr time points. Kinetic flux rate was estimated by taking the product to substrate ratio for identical time points for each group and inter group relative difference in flux rate was compared by dividing the kinetic flux rate of siCAPER with the same of the siControl group. The results were plotted for two different time points (5min and 3 hours respectively) as well.

The Spearman correlation coefficient for each pair of common metabolites between cell line and worm samples was transformed using Fisher’s Z-score transformation, followed by a comparison using the two sample t-test to estimate their significance. The differential analysis was performed separately by comparing both groups of cells to each of the worm RNAi groups. Also the % of total variable pairs which are significant (p<0.01) for four comparisons were also estimated.

**RNA-seq**

The Genomic and RNA Profiling Core first conducted Sample Quality checks using the NanoDrop spectrophotometer and Agilent Bioanalyzer. We then used the NuGEN RNA-Seq and Illumina library preparation system upon manufacturers’ instructions.

**Biostatistical analyses of RNA-seq**

The lengths of the raw reads were about 100 bases. In order to increase the mappability, we trimmed the low-quality bases from both ends of the reads (13 bases from the left end and ~11 bases from the right end). The resulting 76 base pair-ended reads were mapped to the mouse genome (UCSC mm9) using Tophat [10] with NCBI RefSeq genes as the reference and up to two possible mismatches. In order to reduce possible PCR biases, we removed the read duplicates using Samtools rmdup[11]. Transcripts were assembled using Cufflinks v2.0.2 and the raw counts of transcripts were obtained using Cuffdiff with their default parameters[12]. Genes with RefSeq annotation were tested for differential expression using software DESeq [13] and the gene-based transcript counts obtained from Cuffdiff. The Benjamini and Hochberg method was used to control false discovery rate (FDR). At the FDR level of 0.05, 1046 genes out of 22573 genes under test reached statistical significance.

**IPA analyses of RNA-seq and metabolomic data.**

The official gene IDs and fold changes of siCAPER to siControl of the genes at the FDR level of 0.05 were subjected into IPA software to perform core analyses. We showed top functions and pathways presented with high p value or Z score. Metabolomic data were converted into human metabolomic database (HMDB) IDs and subjected into IPA analyses. We showed top functions and pathways presented upon significance of p values.

*C. elegans* **experiments**

*C. elegans* strains and RNAi experiments: C. elegans strains used were rrf-3(pk1426)II, and MW569 (raxl517)[nhx-2p::mCherry;;lgg-1]

[14] were cultured at 20^o^C on nematode NGM agar plates seeded with *Escherichia coli* strain OP50. Strains were obtained from the *Caenorhabditis* Genetics Center (University of Minnesota). Bacterial feeding RNAi experiments were carried out as described [15]. RNAi clone used was sjj Y55F3A_746.f.

Lifespan: Lifespan assays were performed at 20^o^C, as described [15,16]. Briefly, 100 NL2099*rrf-3(pk1426)* II were used per conditions and counted every two days. Animals that escaped from the plate were censored. Survival analyses were performed using the Kaplan Meier method and the significance of difference between two groups calculated using the log rank test. Survival curve were generated using GraphPad Prism 5 for the calculation of mean lifespan and SEM.

Reproduction: Egg numbers were counted from 10 animals daily during their reproductive period.

Respiration assays: Oxygen consumption was measured using Seahorse XF24 (Seahorse Bioscience Inc.) as described. Typically, 300 of 1-day-old animals were recovered from the plates and washed with M9 media and divided into 10 wells of XF24 plates (30 worms per well) and oxygen consumption was measured 6 times followed by 10 μM of FCCP. Respiration rates were normalized to the number of worms in each individual well.

Quantification of ATP levels: Total ATP levels were measured as similar to murine liver cells. Briefly, 30 of 1-day-old, 2-day-old and 3-day-old adult animals were recovered from the plates and washed with M9 media and divided into 5 replicates. Animals were lysed with lysis solution and measures ATP content using luminometer according to manufacturer’s instruction by ATP lite kit (Perkin Elmer).

Quantitative Real-time PCR for gene expression: 500 of 1-day-old adult worms of NL2099*rrf-3(pk1426)* II strain were recovered from NGM plates followed by washing with M9 buffer. Total RNA was prepared using in combination of TRIzol (Invitrogen) and Rneasy kit (Qiagen). 2 μg of RNA was used for reverse transcription. 70X diluted cDNA was used for RT-qPCR which were performed using Step One plus (AB) and SyBr green master mix (AB) with the indicated primers [17].

Lysotracker staining and Fluorescence microscopy: 10 live day 3- old adult worms of NL2099 *rrf-3(pk1426)*II strain fed RNAi bacteria since L1 stage were washed with M9 buffer followed by5min- incubation with Lysotracker (final concentration 1 μM in M2) at room temperature. After incubation, worms were washed with M9 buffer. For autophagic puncta 10 day5-old mCherry::Lgg-1 worms fed RNAi bacteria since L1 stage were subjected to microscopy. Fluorescence microscopy was performed. Animals were paralyzed with 25μM sodium azide and placed on the agarose pad on the glass slide. The GFP filter and RFP filter with EVOS microscopy were employed for imaging lysosensor, *mCherry* ;; Lgg-1 or lysotracker signals from worms at 40X magnification with exposure time of 500 ms for GFP filter, 125ms for RFP filter, respectively. For the high magnitude picture of *mCherry* :: Lgg-1, Day 5 adult worms were mounted on 2% agarose pads containing 0.5% NaN3 as anesthetic on glass microscope slides. Fluorescent images were taken using an Axioplan 2 microscope (Zeiss) connected to an Axioplan MrC camera (Zeiss). Images were quantitated by Image J software (NIH) and statistical analyses were performed with GraphPad software.

**Supplementary References**

1. Kang YK, Jung SY, Qin J, Li C, Tsai SY, et al. (2014) E2/Estrogen receptor/sjogren syndrome-associated autoantigen relieves coactivator activator-induced G1/S arrest to promote breast tumorigenicity. Mol Cell Biol 34: 1670-1681.

2. Putluri N, Shojaie A, Vasu VT, Vareed SK, Nalluri S, et al. (2011) Metabolomic profiling reveals potential markers and bioprocesses altered in bladder cancer progression. Cancer Res 71: 7376-7386.

3. Haschemi A, Kosma P, Gille L, Evans CR, Burant CF, et al. (2012) The sedoheptulose kinase CARKL directs macrophage polarization through control of glucose metabolism. Cell Metab 15: 813-826.

4. Lorenz MA, El Azzouny MA, Kennedy RT, Burant CF (2013) Metabolome response to glucose in the beta-cell line INS-1 832/13. J Biol Chem 288: 10923-10935.

5. Team RDC (2008) R: A language and environment for statistical computing. . Vienna, Austria.

6. Yang YXaJYH (2007) DEDS: Differential Expression via Distance Summary for Microarray Data. R package version 1.30.0. ed.

7. Tibshirani DMWaR (2011) sparcl: Perform sparse hierarchical clustering and sparse k-means clustering. R package version 1.0.2 ed.

8. Warnes GR (2011) gplots: Various R programming tools for plotting data. R package version 2.10.1. ed.

9. Revelle.W. (2013) psych: Procedures for Personality and Psychological Research. psych Version = 1.3.2. ed: Northwestern University, Evanston, Illinois, USA,.

10. Trapnell C, Pachter L, Salzberg SL (2009) TopHat: discovering splice junctions with RNA-Seq. Bioinformatics 25: 1105-1111.

11. Li H, Handsaker B, Wysoker A, Fennell T, Ruan J, et al. (2009) The Sequence Alignment/Map format and SAMtools. Bioinformatics 25: 2078-2079.

12. Trapnell C, Williams BA, Pertea G, Mortazavi A, Kwan G, et al. (2010) Transcript assembly and quantification by RNA-Seq reveals unannotated transcripts and isoform switching during cell differentiation. Nat Biotechnol 28: 511-515.

13. Anders S, Huber W (2010) Differential expression analysis for sequence count data. Genome Biol 11: R106.

14. Melendez A, TallÃoczy Z, Seaman M, Eskelinen E-L, Hall DH, et al. (2003) Autophagy Genes Are Essential for Dauer Development and Life-Span Extension in C. elegans. Science 301: 1387-1391.

15. Houtkooper RH, Mouchiroud L, Ryu D, Moullan N, Katsyuba E, et al. Mitonuclear protein imbalance as a conserved longevity mechanism. Nature 497: 451-457.

16. Wang MC, O'Rourke EJ, Ruvkun G (2008) Fat Metabolism Links Germline Stem Cells and Longevity in C. elegans. Science 322: 957-960.

17. Mouchiroud L, Houtkooper RH, Moullan N, Katsyuba E, Ryu D, et al. (2013) The NAD(+)/Sirtuin Pathway Modulates Longevity through Activation of Mitochondrial UPR and FOXO Signaling. Cell 154: 430-441.
