# Supplementary material for: Correction: CAPER Is Vital for Energy and Redox Homeostasis by Integrating Glucose-Induced Mitochondrial Functions via ERR-α-Gabpa and Stress-Induced Adaptive Responses via NF-κB-cMYC
Source: PLoS Genet. 2015 Jun 15;11(6):e1005316. doi: 10.1371/journal.pgen.1005316 (PMC4468271; doi:10.1371/journal.pgen.1005316)
Supplement: S2 File — (PDF) [file pgen.1005316.s002.pdf]

PLOS | GENETICS

RESEARCH ARTICLE

CAPER Is Vital for Energy and Redox Homeostasis by Integrating Glucose-Induced Mitochondrial Functions via ERR-α-Gabpa and Stress-Induced Adaptive Responses via NF-κB-cMYC

Yun Kyoung Kang[1], Nagireddy Putluri[2,3]*, Suman Maity[2,3]*, Anna Tsimelzon[3,4], Olga Ilkayeva[5], Qianxing Mo[3,6], David Lonard[1], George Michailidis[7], Arun Sreekumar[2,3], Christopher B. Newgard[5], Meng Wang[8,9], Sophia Y. Tsai[1], Ming-Jer Tsai[1], Bert W. O'Malley[1,3]*

1 Department of Molecular and Cellular Biology, Baylor College of Medicine, Houston, Texas, United States of America, **2** Verna and Marrs McLean Department of Biochemistry and Alkek Center for Molecular Discovery, Baylor College of Medicine, Houston, Texas, United States of America, **3** Dan L. Duncan Cancer Center, Baylor College of Medicine, Houston, Texas, United States of America, **4** Lester and Sue Smith Breast Center, Baylor College of Medicine, Houston, Texas, United States of America, **5** Sarah W. Stedman Nutrition and Metabolism Center, Duke University Medical School, Durham, North Carolina, United States of America, **6** Department of Medicine, Baylor College of Medicine, Houston, Texas, United States of America, **7** Department of Statistics, University of Michigan, Ann Arbor, Michigan, United States of America, **8** Huffington Center on Aging, Baylor College of Medicine, Houston, Texas, United States of America, **9** Department of Molecular and Human Genetics, Baylor College of Medicine, Houston, Texas, United States of America

* berto@bcm.edu

CrossMark
click for updates

OPEN ACCESS

Citation: Kang YK, Putluri N, Maity S, Tsimelzon A, Ilkayeva O, Mo Q, et al. (2015) CAPER Is Vital for Energy and Redox Homeostasis by Integrating Glucose-Induced Mitochondrial Functions via ERR-α-Gabpa and Stress-Induced Adaptive Responses via NF-κB-cMYC. PLoS Genet 11(4): e1005116. doi:10.1371/journal.pgen.1005116

Editor: Gregory P. Copenhaver, The University of North Carolina at Chapel Hill, UNITED STATES

Received: July 1, 2014

Accepted: March 3, 2015

Published: April 1, 2015

Copyright: © 2015 Kang et al. This is an open access article distributed under the terms of the Creative Commons Attribution License, which permits unrestricted use, distribution, and reproduction in any medium, provided the original author and source are credited.

Data Availability Statement: All relevant data are within the paper and its Supporting Information files.

Funding: This project was supported by the NIH grants—5R01HD-8188 and 3U19DK062434-11 to BWO; 1R01CA133458 NCI Cancer Center Support Grant (P30CA125123), NCI (AS) and RP120092 from CPRIT (AS PR). This work also received support from the Alkek Foundation. The funders had no role in study design, data collection and analysis, decision to publish, or preparation of the manuscript.

Abstract

Ever since we developed mitochondria to generate ATP, eukaryotes required intimate mito-nuclear communication. In addition, since reactive oxygen species are a cost of mitochondrial oxidative phosphorylation, this demands safeguards as protection from these harmful byproducts. Here we identified a critical transcriptional integrator which eukaryotes share to orchestrate both nutrient-induced mitochondrial energy metabolism and stress-induced nuclear responses, thereby maintaining carbon-nitrogen balance, and preserving life span and reproductive capacity. Inhibition of nutrient-induced expression of CAPER arrests nutrient-dependent cell proliferation and ATP generation and induces autophagy-mediated vacuolization. Nutrient signaling to CAPER induces mitochondrial transcription and glucose-dependent mitochondrial respiration via coactivation of nuclear receptor ERR-α-mediated Gabpa transcription. CAPER is also a coactivator for NF-κB that directly regulates c-Myc to coordinate nuclear transcriptome responses to mitochondrial stress. Finally, CAPER is responsible for anaplerotic carbon flux into TCA cycles from glycolysis, amino acids and fatty acids in order to maintain cellular energy metabolism to counter mitochondrial stress. Collectively, our studies reveal CAPER as an evolutionarily conserved 'master' regulatory mechanism by which eukaryotic cells control vital homeostasis for both ATP and

Competing Interests: The authors have declared that no competing interest exists.

antioxidants via CAPER-dependent coordinated control of nuclear and mitochondrial transcriptomic programs and their metabolisms. These CAPER dependent bioenergetic programs are highly conserved, as we demonstrated that they are essential to preserving life span and reproductive capacity in human cells—and even in *C*. *elegans*.

Author Summary

Energy homeostasis is a vital prerequisite for optimal nutrient utilization and prolonged survival in an environment with fluctuating and frequently scarce food resources. Numerous studies have elucidated the important roles of mitochondrial energy in fasting status but less is known about the role of mitochondria in fed status. Two recent studies elucidated the importance of nutrient-induced mitochondrial functions [1,2] in mammalian longevity, but these studies did not either address how these critical nutrient-induced mitochondrial functions are integrated with nutrient-enhanced antioxidant capacities—nor identify how the carbon and nitrogen balance is maintained. Our study reveals CAPER, as the `first' example of a coregulator nodal integrator which eukaryotes share to orchestrate both nutrient-induced mitochondrial energy metabolism by coactivating ERR-α-Gabpa and stress-induced adaptive metabolic responses via NF- κB/c-Myc; this allows maintenance of carbon-nitrogen balance as well as preservation of life span and reproductive capacity. These metabolic roles for the CAPER coactivator in energy homeostasis are highly conserved and crucial for life span and reproduction in human cells and *C. elegans*.

Introduction

An efficient metabolic adaptation to nutrient status is essential to promote growth, reproduction and to safeguard survival and life span. Because most eukaryotes execute aerobic oxidation through genes encoded by the nuclear genome and the mitochondrial genome, eukaryotes must coordinate their two genomes to efficiently and dynamically regulate energy metabolism. In addition, the inevitable oxidative stress as a cost of mitochondrial oxidative phosphorylation demands that eukaryotes devise safeguards to protect their constituents while they enhance anabolic energy synthesis. Our present study seeks to understand the molecular mechanisms by which eukaryotes sense quantities of nutrients and integrate nutrient signals between the nucleus and mitochondria to maintain energy homeostasis and antioxidant capacities.

CAPER (designated RBM39) is a nuclear receptor coactivator that was shown to facilitate the function of ASC-2 for AP1 and ERα transcription factors [3] and nuclear receptor-dependent alternative splicing [4]. Despite prior indications for a role in cancer cells [5,6], the physiological roles for CAPER in normal cells remained to be established. Here, we uncover CAPER as a previously unknown nodal integrator which eukaryotes use to sense quantities of nutrients and to coordinate their bigenomes; CAPER stimulates mitochondrial activities by coactivating ERR-α-Gabpa to induce nuclear genes and also activates adaptive responses to the increased mitochondrial oxidative stress signals submitted to nucleus by facilitating NF-κB-c-Myc. These roles of CAPER in anabolic energy metabolism and redox capacities are highly conserved in *C. elegans*, in turn preserving life span and reproductive capacity.

Results

Inhibition of nutrient dependent quantitative changes in CAPER suppresses nutrient-dependent cell proliferation and induces autophagy-mediated vacuolization

A high sequence conservation of the CAPER gene among eukaryotes (S1 Table) has indicated its essential core functions shared among eukaryotes. We hypothesized a vital role for CAPER in metabolism because metabolic adaptability is the most pervasive determinant of species survival during evolution. As CAPER transcripts are very abundant in many major metabolic organs including liver [4] and the human CAPER gene is mapped as an expression quantitative trait loci (eQTL) for glucose homeostasis (S2 Table), we initiated our investigations on the metabolic roles for CAPER using nontransformed hepatocytes which maintain many metabolic features of primary hepatocytes [7].

We first asked whether CAPER senses extracellular nutrient signals by examining its quantitative changes upon variation in extracellular glucose levels; mRNA levels of CAPER were found to be increased by glucose in a dose dependent manner, suggesting that CAPER levels reflect quantitative variations in extracellular glucose concentration (Fig 1A). We further examined the roles for CAPER in glucose-dependent cell proliferation by siRNA-mediated inhibition of CAPER expression. Loss of CAPER by siRNA abolished glucose-dependent cell proliferation (Fig 1B), supporting an essential role for CAPER in glucose-dependent cell proliferation.

Our kinetic growth assays showed that the difference in numbers between control and siCAPER transfected cells was obvious from three days after transfection (S1A (i) Fig). By 5 days, the number of siCAPER transfected cells was less than 10% of controls (S1A (i) & (ii) Fig). Western blot analyses confirmed the efficacy of siCAPER which showed undetectable amounts of CAPER protein even 5 days after transfection (S1B (i) Fig) and is consistent with the significantly reduced mRNA levels (S1B (ii) Fig). Our demonstration that three different siRNAs targeting CAPER (S1C Fig) decreased cell proliferation and exogenous CAPER mRNA restored cell proliferation (S1D Fig) excludes the possibility of off-target effect generated by siRNA.

Although there was no significant change in cell numbers after 2 days of siRNA treatment, there was a noticeable sponge-like morphology (Fig 1C upper panel). We found that this vacuolization is caused by increased lysosomal activities without significant changes in lysosomal numbers as shown by (1) distinct patterns of enhanced signals of fluorescent pH indicator-lysosensor that exhibit pH-dependent fluorescence intensity in cells depleted of CAPER but not in control cells (Fig 1C lower panel), (2) complete suppression of vacuolization yet no detectable changes in cell proliferation with lysosomal inhibitors by both chloroquine (CQ) and E64d (Fig 1D) and (3) no significant difference in lysosomal numbers scored by both Lamp1 and Lamp2 as lysosomal markers in both cells (S1E Fig).

Since lysosomal activities are known to be enhanced upon fusion with autophagosomes [8], we further examined whether autophagy is induced in CAPER knocked down cells. We observed increased LC3II, suggesting the induction of autophagy (Fig 1E (i)). Interestingly, like E64d and CQ which block lysosomal degradation of LC3, we observed the accumulation of both LC3I and LC3II in the cells knocked down in CAPER (Fig 1E (ii)). We next determined the effect of CAPER on the autophagic flux. We scored both GFP-LC3 puncta indicating neutral autophagosomes and RFP-LC3 indicating acidic autolysomes; data obtained after knocking down CAPER in cells stably expressing LC3 fused with the tandem fluorescent protein Tf-LC3 are presented (Fig 1F (i) & (ii)). We found increases in both GFP-LC3 puncta and RFP-LC3 puncta from the CAPER knocked-down cells, indicating that depleting CAPER indeed induces autophagy. More prominent RFP signals (Fig 1F (ii) right panel) from the CAPER depleted

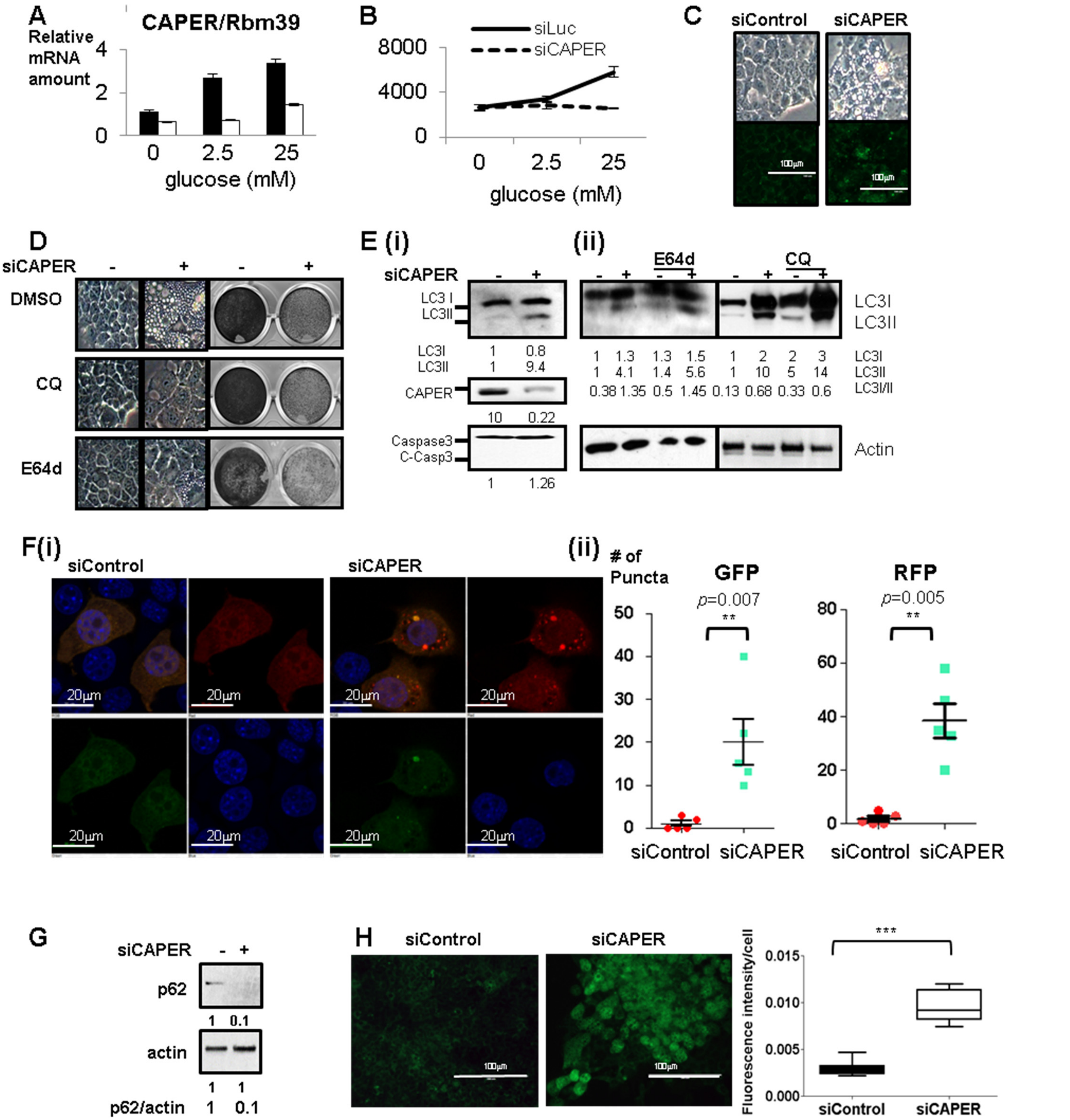

Fig 1. Inhibition of glucose-dependent increased CAPER protein expression suppresses cell proliferation and induces autophagy-mediated vacuolization. A. Glucose dependent increase in CAPER expression. A graph of qRT-PCR showing relative CAPER mRNA levels at 2 days after transfecting each siRNA (black solid bar: control siRNA, white solid bar: siRNA targeting CAPER) from the cells cultured in the media containing the indicated concentrations of glucose. Levels of CAPER mRNA are normalized with the amount of β-2MG as an internal control. Each bar represents the mean value of normalized mRNA levels of CAPER from triplicates with an error bar (SEM). Data were analyzed by t-test using GraphPad software. B. Inhibition of CAPER expression suppresses glucose-dependent cell proliferation. A growth curve showing the decreased cell numbers that were counted at 3 days after

transfecting either siControl (black solid line) or siCAPER (black dotted line). Cells were cultured in the media containing the indicated concentrations of glucose. C. Knockdown of CAPER induces vacuolization with enhanced lysosomal activities. Representative microscopic photos showing the morphology of cells 2 days after transfection of indicated siRNAs (upper panel). Representative microscopic pictures showing cells stained with Lysosensor Green DND-189 (green) 2 days after transfecting with indicated siRNAs (lower panel). Pictures were taken as described in Materials and Methods. D. Representative microscopic pictures showing morphology at 2 days (Left panel) and crystal violet staining pictures showing cell proliferation at 6 days (right panel) after transfection with indicated siRNAs followed by treatment with either DMSO or 10 μM of Chloroquine (CQ) or 10 μM of E-64d. E. (i) Western blot showing that LC3II (an autophagic marker) was induced but not cleaved caspase 3 (an apoptotic marker) in cells treated with siCAPER compared to siControl. Cells were harvested and processed for Western blot analyses 2 days after transfection with the indicated siRNAs. (ii) Western blot showing the efficacy of E64d and CQ on LC3. Cells were harvested and processed for Western blot analyses 2 days after transfection with the indicated siRNAs followed by treatment of E64d and CQ for another 1 day. Band intensity was quantitated by Image J software (NIH). F. A representative microscopic picture (i) and a quantitative graph (ii) showing that RFP-LC3 (red) and GFP-LC3 (green) dots in cells stably expressing tandem fluorescence LC3 fusion proteins (RFP-GFP-LC3) transfected with either siControl or siCAPER. DAPI staining (blue) was used to mark DNA. Pictures were taken 2 days after transfection. A quantitative graph showing the statistical significance of the increase of GFP-LC3 dots (left) and RFP-LC3 dots (right) in cells treated with siCAPER (blue) as compared to cells with siControl (red). Numbers of dots were counted 2 days after transfection with the indicated siRNA. A graph presents numbers of dots counted from the 5 random fields (each point) from three independent experiments. Average (middle bar) and SEM (error bar) are shown. Data were analyzed by t-test using GraphPad software (* $p<0.5$, ** $p<0.01$, *** $p<0.005$). G. Western blot showing lower p62 protein amount in cells knocked down of CAPER than control harvested 2 days after transfection. Actin was served as a loading control. Band intensity was quantitated by Image J software (NIH). H. Fluorescence pictures (left panel) showing carboxy-DCFDA (green) stained cells after transfection with either siControl (left panel) or siCAPER and their corresponding quantitative graphs (right panel) Pictures were taken at “72 hours” after siRNA transfection as described in the experimental procedure (right panel). A scale bar is present. Staining intensity was quantified by Image J software (NIH) and statistical significance was calculated by student’s t-test using GraphPad software.

doi:10.1371/journal.pgen.1005116.g001

cells indicates the presence of more autolysosomes than autophagosomes in the cells. To further confirm the effect of CAPER on autophagic flux, we scored protein levels of p62 that are known to be a marker for autophagic flux [9]. We found that this protein is decreased in the cells depleted of CAPER (Fig 1G). These results indicate an increased autophagic flux upon knocking down CAPER.

To validate the specificity of these phenotypes produced by knock-down of CAPER, we transfected siRNAs targeting PGC-1α and SRC-2 which are known to be involved in aspects of energy metabolism [10,11]. We found that neither knockdown of PGC-1α (S2A, S2B, and S2C Fig) nor SRC-2 (S2D Fig) coactivators could recapitulate the phenotypes observed when CAPER expression was depleted. Exogenous expression of SRC-2 could not complement effects of the knock-down of CAPER (S2E Fig). To investigate whether these phenotypes are specific to a cell type, we tested primary hepatocytes (S2F Fig) and hepatoma cells (S2G Fig) which recapitulate the phenotypes.

As indicated by undetectable cleaved caspase 3 which is an executioner of apoptosis (Fig 1E (i)), we did not observe significantly enhanced apoptosis in the cells knocked down in CAPER compared to control (S3A Fig). Our demonstration that knockdown of CAPER induces autophagy but not apoptosis hints that depleting CAPER resembles the mild and reversible insults in which p53 and p21 are known to be involved [12]. Cell cycle arrest at both G1/S and G2/M (S3B Fig) with a dramatic induction of p21 and a modest increase of p16 (S3C Fig) starting from 36 hours of posttranfection, prior to autophagy, suggests that knockdown of CAPER arrests cell proliferation prior to autophagy. This explains our observation that lysosomal inhibitors fail to restore cell proliferation (Fig 1D). These results, together with undetectable changes in the amount of p53 transcripts (S3C Fig), fit the previously suggested model of cellular survival responses to reversible stress [12].

As one of the key responses required for cell survival is the Nrf-2 mediated antioxidant pathway known to be induced by p21 [13], we examined the expression of Nrf-2 and cellular ROS levels in cells depleted of CAPER. Transient lower expression of Nrf2 (S3C Fig) at 48 hours of posttransfection and prominent staining of (5-(and-6)-Carboxy-2',7'-Dichlorofluorescein Diacetate (carboxy-DCFDA) after “3 days” of posttransfection (Fig 1H) indicates strong oxidative stress in cells depleted of CAPER. This suggests that cells without CAPER fail to induce antioxidant defense responses that are crucial for cell survival. Taken together, these

results suggest that the initial responses to CAPER deficiency resemble ones noted in reversible stress which arrests the cell cycle followed by induction of autophagy. However, lack of CAPER ultimately leads to oxidative stress resulting in irreversible loss of cellular survival capacities.

CAPER coactivates ERR-α to increase glucose-dependent mitochondrial respiration by regulating glucose-induced nuclear and mitochondrial transcription

Because one of the primary causes of cellular ROS is dysfunctional mitochondria, we examined mitochondrial superoxide [14]. Strong induction of mitochondrial superoxide in cells depleted of CAPER at 24 hours of posttransfection (Fig 2A) suggests that dysfunctional mitochondria are the potential cause of the arrested cell cycle [15] and induction of autophagy [14]. A decreased oxygen consumption rate (OCR) measured by a XF24 analyzer (Sea horse bioscience) in cells depleted of CAPER further supports that malfunctional mitochondria exist in cells depleted of CAPER (Fig 2B). Because the primary source of mitochondrial superoxides is electron transport chain (ETC) complexes, we measured the amount of transcripts of ETC complexes. Although no significant changes in mitochondrial biogenesis were observed by determining the ratio of mitochondrial DNA (mtND2) to nuclear DNA (cyclophilin) (Fig 2C (i)) and the intensity of Mitotracker Red (Fig 2C (ii))) in cells depleted of CAPER, all examined mitochondrial transcripts and nuclear encoded mitochondrial transcription machineries such as Gabpa [16] were significantly decreased (Fig 2D). Consistent with a decreased amount of Gabpa transcripts, other targets of Gabpa such as complex I components were reduced in cells knocked down in CAPER (Fig 2D).

Because ERR-α is a known upstream activator for Gabpa transcription, we tested whether CAPER coactivates ERR-α to regulate expression of mitochondrial transcription machineries. CAPER interacts with ERR-α (Fig 2E (i)) and coactivates it to enhance the promoter of Gabpa (Fig 2E (ii)). In addition, CAPER coactivates Gabpa itself in a transient transfection assay (Fig 2E (ii)). The presence of CAPER on the promoters of Gabpa and Tfam shown by chromatin immunoprecipitation (ChIP) assays (Fig 2E (iii)) indicates that CAPER directly regulates Gabpa as well as its target Tfam.

Because CAPER is induced by glucose, we asked whether CAPER is required for glucose-dependent mitochondrial transcription. We found that indeed CAPER is necessary for glucose-dependent induction of nuclear encoded mitochondrial transcription machineries (Fig 2F). To assess the functional relevance of glucose-induced mitochondrial transcription, we measured oxygen consumption rate (OCR) using the XF24 analyzer with or without glucose. Our demonstration of the necessity of CAPER for Carbonyl cyanide 4-(trifluoromethoxy)-phenylhydrazone (FCCP)-induced mitochondrial maximal respiratory capacities (from B to C) in the presence of glucose (Fig 2G (i), but not without glucose (Fig 2G (ii)), supports the requirement of CAPER for glucose dependent mitochondrial function. Taken together, our results suggest a crucial regulatory role for CAPER in glucose-induced mitochondrial function.

Inhibition of CAPER induces nuclear transcriptional changes resembling retrograde responses and disturbs carbon-nitrogen balance

Consecutive treatments of oligomycin followed by FCCP dissipate the mitochondrial proton gradient leading to depolarized mitochondria. Depolarized mitochondria are known to signal to the nucleus to induce adaptive responses known as `retrograde responses'[17]. However, our data suggests that CAPER deficient cells are not capable of inducing compensatory respiration to combat mitochondrial insults (Fig 2G (i)). In addition, the fact that inhibiting autophagy which is a compensatory survival response does not restore cell proliferation (Fig 1D)

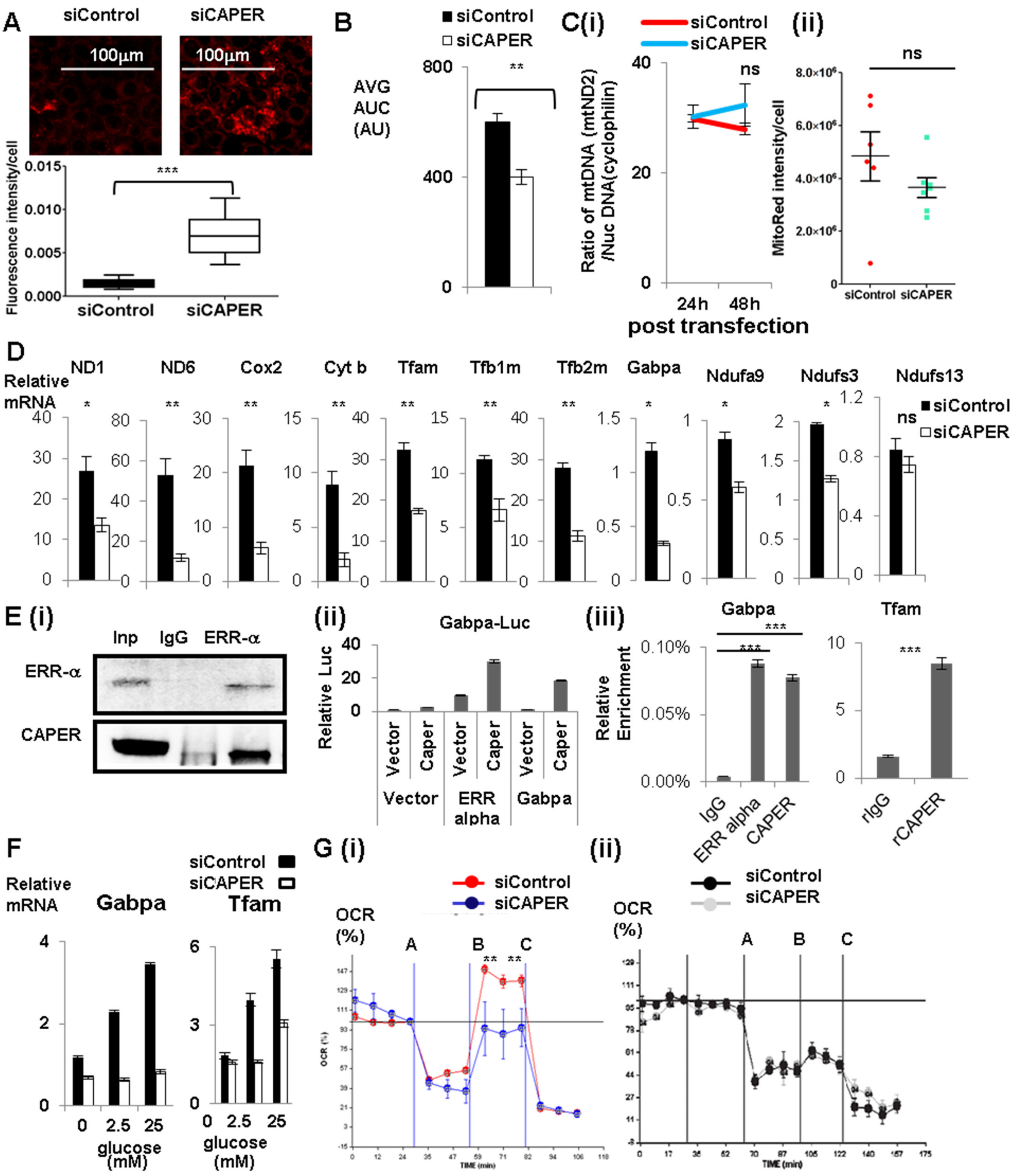

Fig 2. CAPER increases glucose-dependent mitochondrial respiration by regulating glucose-induced mitochondrial transcription via ERR α -Gabpa. A. Fluorescence pictures (upper panel) showing MitoSox (red) stained cells after transfection with either siControl (left panel) or siCAPER (right

panel) and their corresponding quantitative graphs (lower panel). Pictures were taken at 24 hours after siRNA transfection. A scale bar is present. Staining intensity was quantified by Image J software and statistical significance was calculated by student's t-test using GraphPad software. B. A graph presenting the area under the curve (AUC) of basal OCR from cells 24 hours after transfection with either siControl (black) or siCAPER (white) with assay media containing 50mM pyruvate. C. Graphs showing the ratio of mitochondrial DNA copy number (mtND2) to nuclear DNA copy number (cyclophilin) (i) and the intensity of Mitotracker Red (ii). Cells transfected with siCAPER (blue) are compared with siControl (red). Genomic DNAs were isolated from cells harvested at the indicated time after transfection followed by qPCR to quantitate relative ratio of DNA copy numbers by measuring amount of mitochondrial ND2 and nuclear cyclophilin. D. Graphs showing lower mRNA levels of mitochondrial genes and mitochondrial transcription machinery and representative Gabpa target genes encoding ETC complex I in cells transfected with siCAPER (white) than cells with siControl (black). E. (i) Immunoprecipitation assays showing that endogenous CAPER and endogenous ERR α interact with each other. Cleared whole cell extracts in BC180-0.05 (BC buffer containing 180mM KCl with 0.05% of NP-40) from AML 12 cells were subjected to immunoprecipitation with either a rabbit IgG or anti-ERR α antibody. After 4 hours of binding, protein A/G (Santa Cruz) beads were added for another hour. Bound proteins were washed 5 times with BC180-0.1 (BC buffer containing 180mM KCl with 0.1% of NP-40) followed by acid elution using glycine (pH2.5). Elutes were subjected to SDS-PAGE to score the presence of CAPER by Western blot. (ii) Luciferase reporter assays showing that CAPER coactivates ERR αand Gabpa to enhance luciferase reporter activity from the Gabpa promoter. Luciferase assays were performed 48 hours after transfection as described in the Materials and Methods section. (iii) Chromatin immunoprecipitation assay showing that CAPER is present on the ERR α binding sites in Gabpa promoters and the Gabpa binding sites in the Tfam promoter. The procedures were essentially as described in the Materials and Methods. F. qRT-PCR revealed that CAPER and its targets are regulated by glucose in a dose dependent manner. Glucose-dependent induction of target genes were diminished with siCAPER (white) compared to siControl (black). 6 hours after transfection, cells were changed into glucose-free media containing the indicated concentration of glucose for 48 hours. RNAs were extracted and subjected to qRT-PCR analyses as described in the Materials and Methods section. G. (i) A Graph showing real time measurements of OCR of cells treated with either siControl (red) or siCAPER (blue) containing glucose using a XF analyzer in the presence of consecutive treatments with an ATPase inhibitor, oligomycin (A), followed by a protonophore, carbonyl cyanide 4-trifluoromethoxy phenylhydrazone (B), and then lastly by a complex III inhibitor, Antimycin A (C). (ii) A Graph showing real time measurements of OCR of cells treated with either siControl (black) or siCAPER (gray) without glucose using a XF analyzer in the presence of consecutive treatments with an ATPase inhibitor, oligomycin (A), followed by a protonophore, carbonyl cyanide 4-trifluoromethoxy phenylhydrazone (B), and then lastly by a complex III inhibitor, Antimycin A (C).

doi:10.1371/journal.pgen.1005116.g002

supports that CAPER is required for these compensatory responses to be effective. To examine global nuclear responses in CAPER deficient cells, we analyzed the cellular transcriptome upon knock-down of CAPER. Our unbiased transcriptomal analysis revealed differential expression of 1047 genes that are equivalent to about 5% of the entire mouse transcriptome upon knock-down of CAPER. GO analyses for physiological functions revealed strong enrichment of genes for organismic survival (Fig 3A (i)). GO analyses for molecular functions indicated enrichment of genes for protein metabolism including proteolysis and responses to chemical stimuli such as reactive oxygen species (Fig 3A (ii) and S3 Table). Kyoto Encyclopedia of Genes and Genomes (KEGG) analyses revealed the enrichment of genes important for Nicotinamide adenine dinucleotide (NAD^+) in both redox balance and energy metabolism (S3 Table). Finally, Ingenuity Pathway Analyses (IPA) using significantly altered genes revealed the NF-κB pathway to be highly enriched in CAPER dependent genes (Fig 3B). Overall, our results suggest that the CAPER dependent transcriptome includes genes involved in retrograde response-mediated metabolic reactions.

Dysfunctional mitochondrial ETCs cause a truncated TCA cycle leading to a redirection of cellular metabolites to stimulate carbon flux into the TCA cycle. We next profiled [18,19] 45 representative cellular metabolites involved in glucose metabolism, amino acid and palmitate oxidation that are known to be involved in retrograde responses; 24 of 45 (53.3%) metabolites at 24 hours were identified based on analysis by Benjamini, Hochberg corrected $p < 0.05$ (Fig 3C and S4 Table). In CAPER depleted cells, the most significantly upregulated metabolites were amino acids, as our transcriptomal analyses indicated. Conversely, downregulated metabolites were primarily involved in glycolysis (6 metabolites) and the TCA cycle (5 metabolites) with the exception of phosphoenolpyruvate (PEP) that is a glycolytic metabolite (Fig 3C and S4 Table). The fact that most reduced metabolites contain only carbon, unlike the increased amino acids containing both carbon and nitrogen (Fig 3C), suggests an imbalance of carbon-nitrogen metabolites in cells depleted of CAPER. To determine relationships among metabolites, we generated a Spearman's correlation matrix of all pairwise comparisons among individual metabolites using the log-transformed data. Unsupervised hierarchical clustering revealed two major "hot spots" of correlated metabolites ($r>0.7$) at 24 hours (Fig 3C and S4 Table);

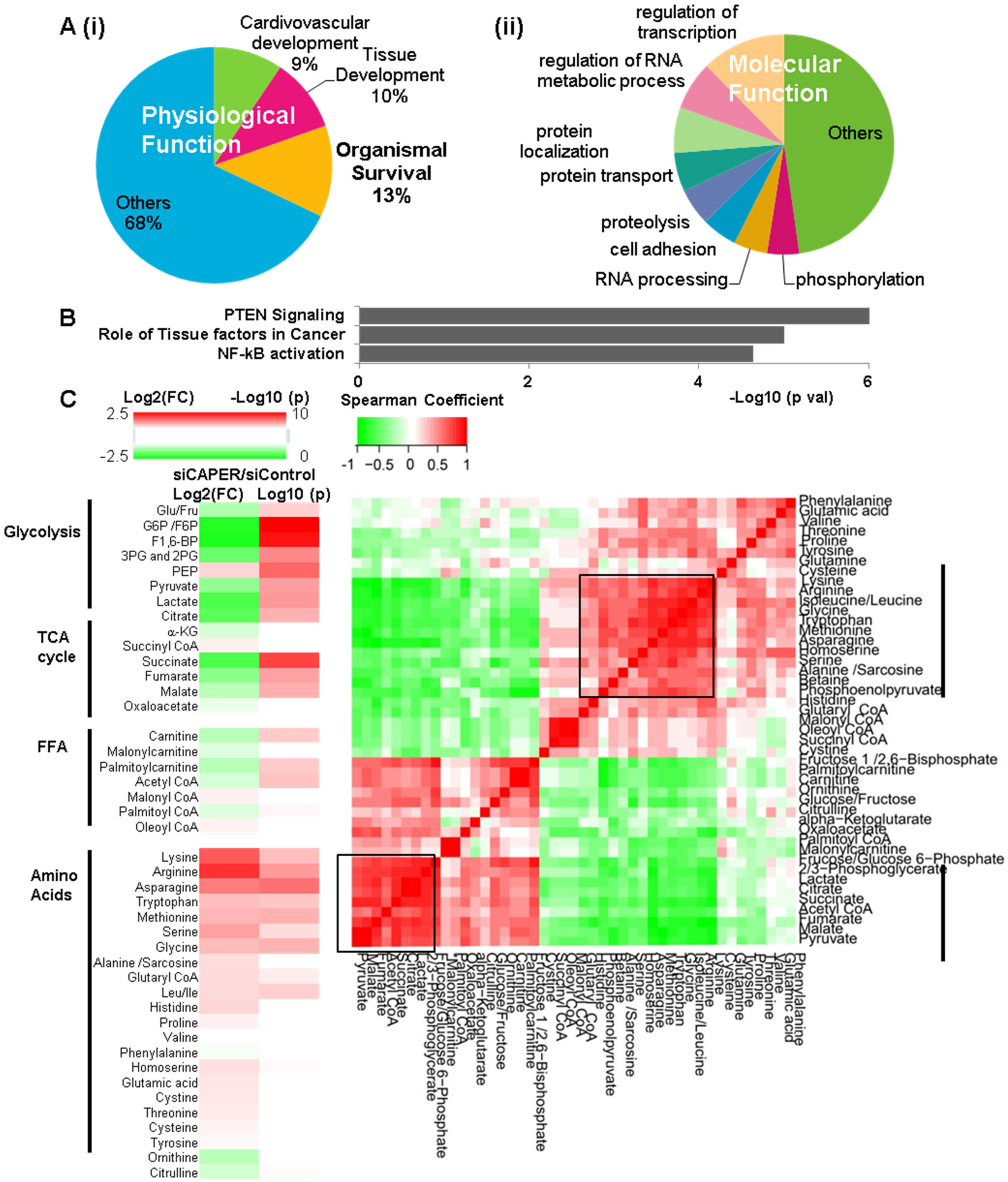
A (i)
Physiological Function
Cardiovascular development 9%
Tissue Development 10%
Organismal Survival 13%
Others 68%
(ii)
Molecular Function
regulation of transcription
regulation of RNA metabolic process
protein localization
protein transport
proteolysis
cell adhesion
RNA processing
phosphorylation
Others
B
PTEN Signaling
Role of Tissue factors in Cancer
NF-kB activation
0
2
4
6
-Log10 (p val)
C
Log2(FC)
-Log10 (p)
2.5
-2.5
10
0
Spearman Coefficient
-1 −0.5 0 0.5 1
siCAPER/siControl
Log2(FC) Log10 (p)
Glycolysis
Glu/Fru
G6P /F6P
F1,6-BP
3PG and 2PG
PEP
Pyruvate
Lactate
TCA cycle
Citrate
α-KG
Succinyl CoA
Succinate
Fumarate
Malate
Oxaloacetate
FFA
Carnitine
Malonylcarnitine
Palmitoylcarnitine
Acetyl CoA
Malonyl CoA
Palmitoyl CoA
Oleoyl CoA
Amino Acids
Lysine
Arginine
Asparagine
Tryptophan
Methionine
Serine
Glycine
Alanine /Sarcosine
Glutaryl CoA
Leu/Ile
Histidine
Proline
Valine
Phenylalanine
Homoserine
Glutamic acid
Cystine
Threonine
Cysteine
Tyrosine
Ornithine
Citrulline
Phenylalanine
Glutamic acid
Valine
Threonine
Proline
Tyrosine
Glutamine
Cysteine
Lysine
Arginine
Isoleucine/Leucine
Glycine
Tryptophan
Methionine
Asparagine
Homoserine
Serine
Alanine /Sarcosine
Betaine
Phosphoenolpyruvate
Histidine
Glutaryl CoA
Malonyl CoA
Oleoyl CoA
Succinyl CoA
Cystine
Fructose 1 /2,6-Bisphosphate
Palmitoylcarnitine
Carnitine
Ornithine
Glucose/Fructose
Citrulline
alpha-Ketoglutarate
Oxaloacetate
Palmitoyl CoA
Malonylcarnitine
Frucose/Glucose 6-Phosphate
2/3-Phosphoglycerate
Lactate
Citrate
Succinate
Acetyl CoA
Fumarate
Malate
Pyruvate

Fig 3. CAPER inhibition reprograms nuclear transcriptomes and metabolomes resembling retrograde responses. A. Pie charts showing the IPA results of top physiological functions (i) and molecular functions (ii) enriched in CAPER-dependent transcriptomes. Each section represents the percentage of the genes in the indicated functional category out of entire CAPER-dependent transcriptomes. B. A graph showing the IPA results of top canonical pathways enriched in CAPER-dependent transcriptomes. Each bar represents $\log_{10}$ (p value) of each functional category out of the entire CAPER-dependent transcriptomes. C. Depletion of CAPER disturbs cellular carbon and nitrogen balance. A heat map (left panel) showing altered metabolites by Log_2 (FC: fold change) (left colum-24 hour samples) and by Log_{10}(p values) (p values adjusted for multiple comparisons using Benjamini-Hochberg method) (right column). Scale of red (positive values) and green (negative values) are represented as a color code on top of the figure. A matrix (right panel) showing the correlation distance calculated by Spearman's correlation analysis among the metabolites from cells 24 hours after transfection with siControl and siCAPER. Scale of red (positive values) and green (negative values) are represented as a color key on top of the figure.

doi:10.1371/journal.pgen.1005116.g003

these two groups corresponded to: (1) amino acids and PEP and (2) metabolites in glycolysis, fatty acid oxidation and the TCA cycle. The results suggest a CAPER dependent maintenance of carbon metabolites by coordinating glycolysis, fatty acid oxidation and the TCA cycle.

CAPER coactivates NF-κB to activate a Myc network

To identify transcriptional changes that correlate with the metabolic phenotypes in cells knocked down by CAPER, we sought common regulators associated with both significantly changed transcriptomes and metabolomes. Unbiased IPA revealed a common regulator: c-Myc (Fig 4A). We found that c-Myc is a downstream target of CAPER as shown by (1) lower transcripts of c-Myc in cells knocked down by CAPER (Fig 4B (i)) and (2) CAPER-mediated activation of NF-κB-dependent c-Myc promoter activity in a transfection assay (Fig 4B (ii)). Our ChIP assays revealed the presence of both CAPER and NF-κB on their corresponding transcription factor binding sites in the c-Myc promoter, substantiating c-Myc and as a direct target of CAPER (Fig 4B (iii)). These results establish CAPER as an upstream regulator of the c-Myc gene by virtue of its coactivation of NF-κB. To investigate the functional relevance of c-Myc in CAPER deficiency, we overexpressed c-Myc in cells knocked down by CAPER. C-Myc overexpression partially enhanced cell proliferation as shown crystal violet staining (Fig 4B (iv)) but did not abolish vacuolization and autophagy as shown by western blot scoring LC3 (Fig 4B (v)).

To further substantiate the roles for CAPER in Myc dependent transcriptional reprogramming of cellular metabolisms, we analyzed genes dependent on both CAPER and c-Myc. About 20% of CAPER-dependent genes also are categorized as c-Myc dependent (Fig 4C (i)). GO analyses with these common genes show functional association with responses to reactive oxygen species and protein metabolisms; (S3 Table), in particular, glutamine metabolism and urea cycle amino acid metabolism, metabolisms resembling those involved in yeast retrograde response. These pathways were supported by analyses showing strong correlations of pairs of pyruvate to malate, citrate to lactate, carnitine to palmitoyl carnitine among metabolomes (S4 Table). These data suggest that CAPER regulates the pathway linking palmitate oxidation and glutaminolysis to provide Acetyl CoA and pyruvate into TCA cycle (as shown in Fig 4C (ii)). Our gene expression assays confirmed the significantly reduced expression of genes which are key enzymes mediating glutamine entry to the TCA cycle (Fig 4C (i)); we confirmed the lower enzymatic activities as shown by the lower ratio of product to substrate in CAPER knocked down cells versus controls (S4 Fig). In addition, we found an altered amount of the enzymes arginosuccinate lyase (Asl) which contributes to nitrogen recycling and fumaratase (Fh1) that mediates anaplerotic pathways connecting the urea cycle and the TCA cycle via aspartate and fumarate (Fig 4C (i) & (ii)). Our demonstration of a lower basal OCR with glutamine as a sole fuel source in cells deprived of CAPER (Fig 4D) further substantiates the inefficient utilization of glutamine as an alternative carbon source in these cells. Taken together, these data establish a role for CAPER in amino acid-mediated anaplerotic carbon flux.

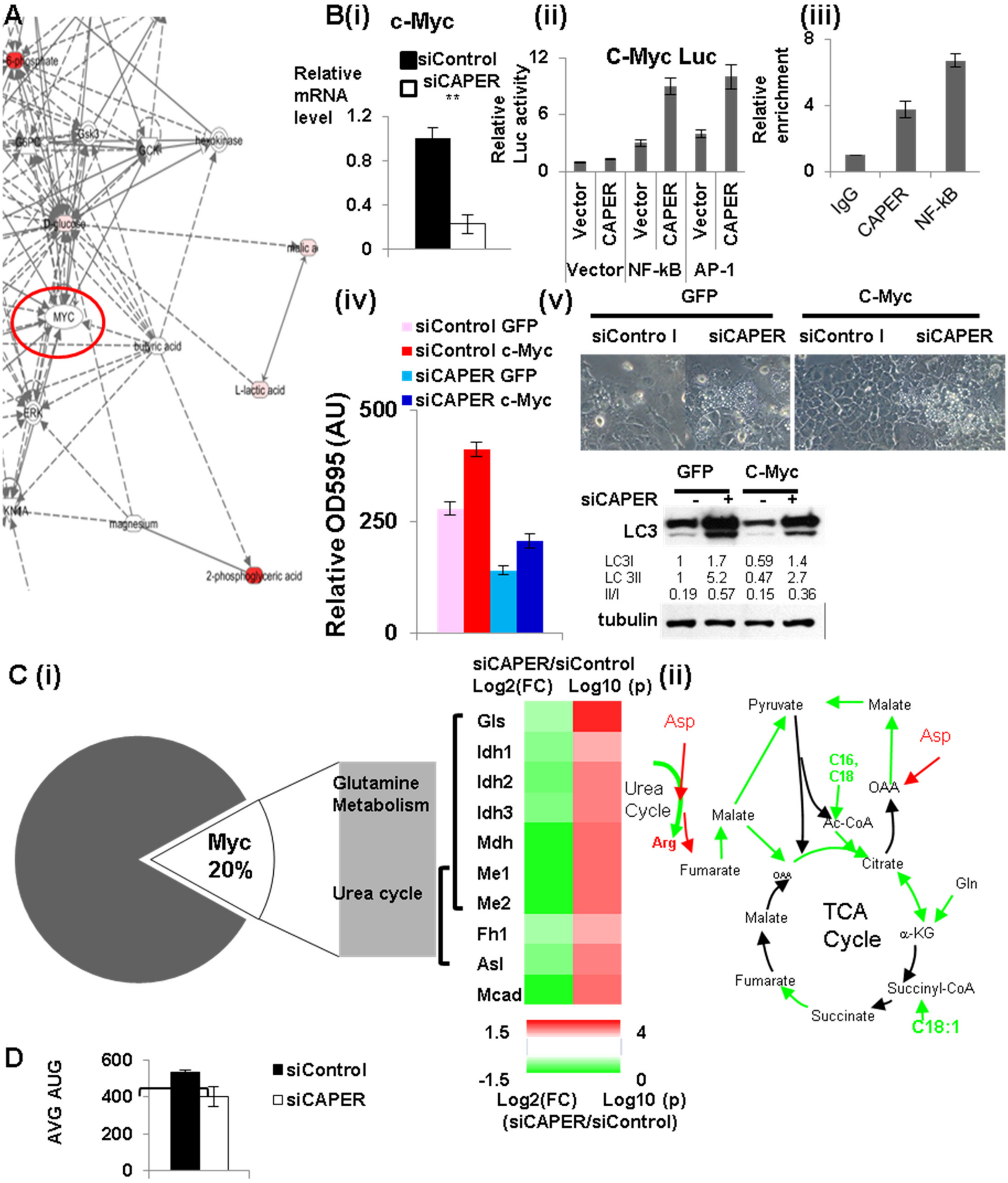
A
MYC
hexokinase
D-glucose
malic a
butyric acid
L-lactic acid
ERK
magnesium
2-phosphoglyceric acid
B(i) c-Myc
Relative mRNA level
siControl
siCAPER
**
(ii)
C-Myc Luc
Relative Luc activity
Vector CAPER Vector CAPER Vector CAPER
Vector NF-kB AP-1
(iii)
Relative enrichment
IgG CAPER NF-kB
(iv)
siControl GFP
siControl c-Myc
siCAPER GFP
siCAPER c-Myc
Relative OD595 (AU)
(v)
GFP
C-Myc
siContro l siCAPER siContro l siCAPER
siCAPER
LC3
LC3I 1 1.7 0.59 1.4
LC 3II 1 5.2 0.47 2.7
II/I 0.19 0.57 0.15 0.36
tubulin
C (i)
Myc 20%
Glutamine Metabolism
Urea cycle
siCAPER/siControl
Log2(FC) Log10 (p)
Gls
Idh1
Idh2
Idh3
Mdh
Me1
Me2
Fh1
Asl
Mcad
Log2(FC) Log10 (p)
(siCAPER/siControl)
(ii)
Pyruvate
Malate
Asp
C16, C18
OAA
Urea Cycle
Arg
Fumarate
Ac-CoA
Citrate
Gln
TCA Cycle
α-KG
Succinyl-CoA
Succinate
C18:1
D
AVG AUG
siControl
siCAPER

Fig 4. CAPER activates c-Myc leading to activating genes involved in amino acid mediated anaplerosis. A. A gene-metabolite network showing the IPA results to identify upstream regulators. B. (i) QRT-PCR analyses showing that the mRNA levels of c-Myc are reduced in cells transfected with siCAPER (white) compared to ones with siControl (black). (ii) Luciferase reporter assays showing that CAPER coactivates NF-κB to enhance luciferase reporter activity from the c-Myc promoter. Luciferase assays were performed 48 hours after transfection as described in the Materials and Methods section. (iii) Chromatin immunoprecipitation assay showing that CAPER is present on the NF-κB binding sites in the c-Myc promoters. A graph presents results from crystal violet staining (iv) and microscopic pictures of cell morphology (v; upper panel) and LC3 western blot (v; lower panel) at 2 days after infection with adenovirus encoding either GFP or c-Myc into cells transfected with the indicated siRNAs. C. (i) A heat map (right panel) showing the relative amount of the enzymes in anaplerotic pathways, analyzed by qRT-PCR by Log_2 (FC: fold change) (left colum-24 hour samples) and by Log_{10}(p values) (p values adjusted for multiple comparisons using Benjamini-Hochberg method) (right column). Scale of red (positive values) and green (negative values) are represented as a color code on bottom of the figure. (ii) A schematic diagram of the CAPER dependent anaplerotic pathways. D. A graph showing average (AUC) of OCR from cells treated with either siControl (black) or siCAPER (white) with a XF analyzer using assay media containing only glutamine. Average of raw OCR values from 9 replicates was plotted and error bars represent SEM (* $p<0.05$).

doi:10.1371/journal.pgen.1005116.g004

CAPER coordinates glucose-derived carbon flux into glycolysis through PPP for glutathione antioxidant capacities and glycolysis through the TCA cycle leading to glucose-induced NADH production

Induction of glycolysis, known as the Warburg effect, is the best example of a compensatory response to mitochondrial dysfunction. Our metabolomic profiling revealed coordinately reduced carbon metabolites involved in glycolysis and the TCA cycle in cells knocked down in CAPER, suggesting that CAPER coordinates glucose derived carbon flux into the TCA cycle. We examined glucose-derived carbon flux into metabolites that are highly associated in the first cluster (shown in the square in Fig 3C) using a stable isotope tracer, [U-13C6]-D-glucose by metabolite flux analyses. Comparable glucose uptake in both cells were shown by a similar amount of total [U-13C6]-D-glucose detected in cells treated with both siRNAs (Fig 5A). Despite the significant reductions in their steady state levels (Fig 3C), the most (>99%) F1/2, 6-BPs was C13 labeled as M+6 during 3 hours (Fig 5A). This suggests that reduced steady state levels of F1/2, 6-BP are likely due to another pathway. This, together with a transient increase in C13 populations of G6P/F6P at 1 hour with the significantly lower C13 populations of G6P/F6P at 3 hours in CAPER depleted cells, suggests a reduced carbon shuffling between G6P/F6P and the pentose phosphate pathway (PPP) in cells depleted of CAPER. Because of the reduced carbon flux into PPP from G6P/F6P, a transient accumulation of G6P/F6P was noted in our data (Fig 5A). This accumulated G6P/F6P could accelerate the alternative carbon flux into F1/2, 6-BP as seen from flux data (S4 Fig).

As PPP is involved in supplying these metabolites, the reduced carbon flux into PPP suggests lower carbon supply to generate glutathione antioxidants in cells depleted of CAPER. Because cells depleted of CAPER are under oxidative stress due to dysfunctional mitochondria, cells should enhance flux into PPP to generate more glutathione antioxidants to resolve harmful oxidative stress. However, our flux analyses, together with lower ratio of glutathione (GSH) to glutathione disulfide (GSSG) in cells knocked down by CAPER (Fig 5B (i)) suggests that cells depleted of CAPER are not capable to enhance glutathione antioxidants. These results also are supported by our transcriptomic data revealing an enrichment of genes for responses to reactive oxygen species. Our demonstration of suppression of GSH/GSSG by 2-deoxyglucose (2-DG) (Fig 5B (ii)) supports the relevance of glucose-derived carbon flux in GSH maintenance. Conversely, the increased GSH upon exogenous addition of F1/2, 6-BP (Fig 5B (ii)) which recycles carbon flux into glycolysis further supports that glucose-derived carbon flux is important to maintain GSH/GSSG in CAPER deficient cells.

In addition, complete suppression of vacuolization induced in CAPER deficient cells by supplying N-acetyl cysteine (NAC) which is GSH precursor (Figs 5C and S5A) or F1/2, 6-BP (Figs 5D and S5A) strongly supports that CAPER is required to suppress ROS-induced autophagy-mediated vacuolization by increasing carbon flux to supply glutathione antioxidants (Fig 5C).

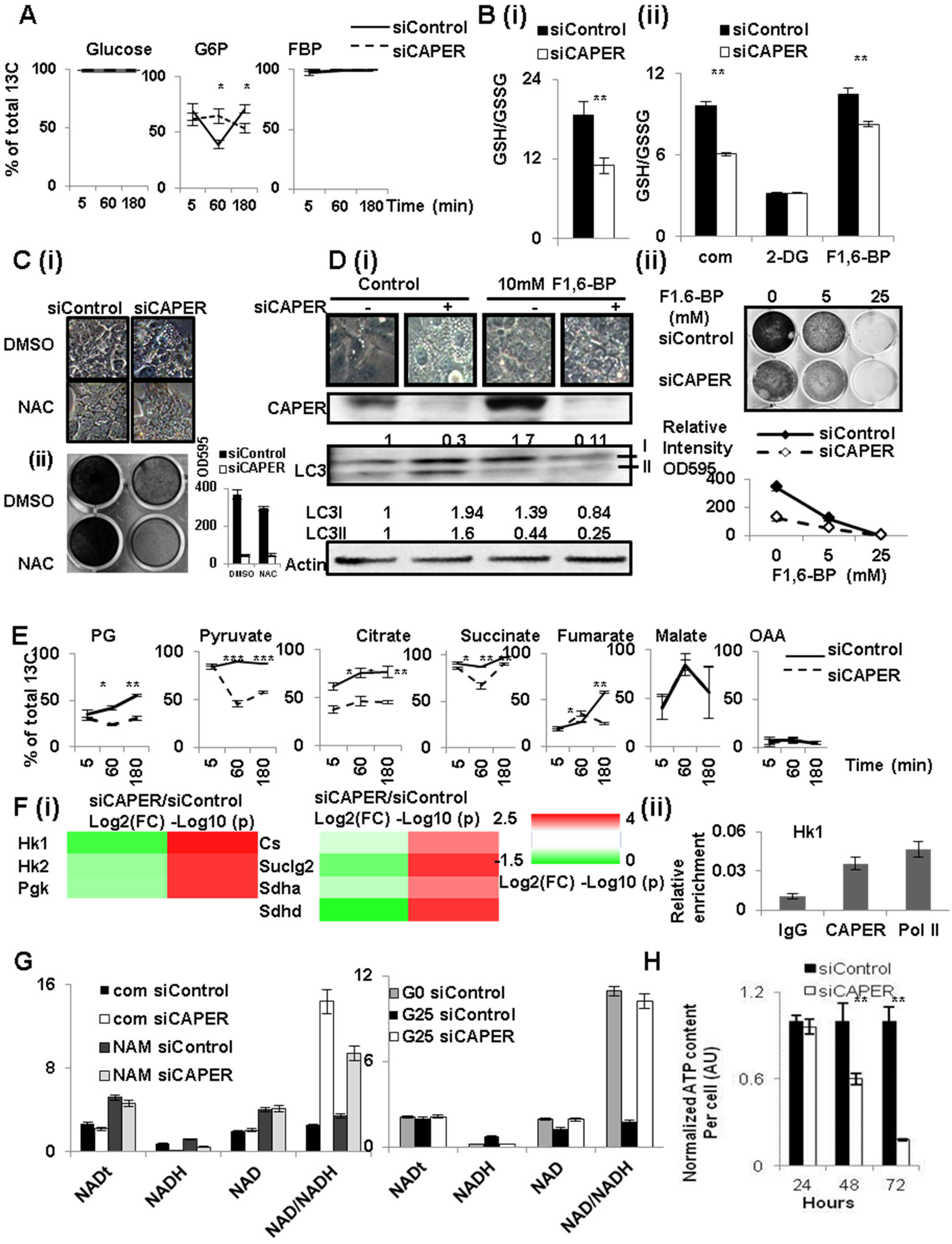

A
Glucose
G6P
FBP
siControl
siCAPER
% of total 13C
Time (min)
B (i)
(ii)
GSH/GSSG
com
2-DG
F1,6-BP
C (i)
siControl siCAPER
DMSO
NAC
(ii)
OD595
D (i)
Control
10mM F1,6-BP
siCAPER
CAPER
LC3
LC3I
LC3II
Actin
F1.6-BP (mM)
Relative Intensity OD595
F1,6-BP (mM)
E
PG
Pyruvate
Citrate
Succinate
Fumarate
Malate
OAA
F (i)
siCAPER/siControl
Log2(FC) -Log10 (p)
Hk1
Hk2
Pgk
Cs
Suclg2
Sdha
Sdhd
(ii)
Relative enrichment
IgG
CAPER
Pol II
G
com siControl
com siCAPER
NAM siControl
NAM siCAPER
G0 siControl
G25 siControl
G25 siCAPER
NADt
NADH
NAD
NAD/NADH
H
Normalized ATP content Per cell (AU)
Hours

Fig 5. CAPER deficiency decreased carbon flux into glycolysis and TCA cycles leading to defective compensatory mitochondrial respiratory responses. A. Glucose-derived carbon incorporation into Glucose, G6P/F6P and F1/2, 6-BP in cells depleted of CAPER. A graph showing the average percentage of ^{13}C labeled metabolites from three biological replicates of cells transfected with siCAPER (dotted line) and ones in cells with siControl (solid line). Cells were harvested at 24 hours after transfection followed by the procedures described in Materials and methods. Error bars represent SEM. B. Graphs showing the ratio of GSH/GSSG from the cells after transfection with either siControl (black solid) or siCAPER (white) in the absence (i) or the presence (ii) of the indicated treatment. A bar represents the mean value of relative GSH/GSSG from triplicate with an error bar (SEM). Data were analyzed by t-test using GraphPad software. C. A microscopic image shows the morphology (i) and crystal violet staining indicating the effect on the proliferation (ii) of cells transfected with either siControl or siCAPER followed by treating with either DMSO or NAC treatments for 24 hours. D. (i) A microscopic image (upper panel) showing the morphology and western blot (lower panel) scoring LC3 isoforms of cells transfected with either siControl or siCAPER followed by treating with either DMSO or 10 mM F1, 6-BP treatments for 24 hours. (ii) A microscopic picture (upper panel) and its corresponding quantitative graph (lower panel) of crystal violet staining shows the effect on the proliferation of cells transfected with either siControl or siCAPER followed by treating with either DMSO or 10 mM F1, 6-BP for 24 hours. E. Decreased glucose-derived carbon incorporation into the shown metabolites in cells depleted of CAPER. A graph showing the average percentage of ^{13}C labeled metabolites from three biological replicates of cells transfected with siCAPER (dotted line) and ones in cells with siControl (solid line). Cells were harvested at 24 hours after transfection followed by the procedures described in Materials and methods. Error bars represent SEM. F. (i) A heat map (right panel) showing the relative amount of the indicated enzymes analyzed by qRT-PCR by Log_2 (FC: fold change) (left colum-24 hour samples) and by Log_{10}(p values) (p values adjusted for multiple comparisons using Benjamini-Hochberg method) (right column). Scale of red (positive values) and green (negative values) are represented as a color code on bottom of the figure. (ii) ChIP assays showing the presence of CAPER on the c-Myc binding sites of the promoter of Hk1. G. Graphs showing amount of total NAD (NADt), NAD, NADH in the cells transfected with siControl and siCAPER followed by treating NAM (left) or glucose (right). G0 and G25 indicate that cells were cultured in media containing either 0 or 25mM glucose, respectively. Average of raw values from 3 replicates was plotted and error bars represent SEM. H. A graph showing that ATP content per cell at the indicated time after transfection with siCAPER (solid white) compared to siControl (solid black).

doi:10.1371/journal.pgen.1005116.g005

Comparable mitochondria numbers regardless of the presence of CAPER with or without NAC (Figs 2C and S5C) further support our previous observation that CAPER deficiency does not result in change in mitochondrial number per se. These results substantiate that supplying NAC or F1/2, 6-BP is not sufficient to complement CAPER deficiency.

The drastically reduced C13 populations of glycolysis and TCA metabolites including phosphoglycerate (2/3-PG), pyruvate, citrate, succinate and fumarate in CAPER depleted cells indeed demonstrates a significant role for CAPER in synchronized glucose-derived carbon flux through the TCA cycle (Fig 5E). In addition, no detectable change in glucose-derived C13 carbon incorporation of malate, together with a reduced incorporation rate of fumarate from succinate, suggests that CAPER-dependent glucose-derived carbon incorporation into TCA cycle stops at the steps connecting with ETCs. Because impaired expressions of mitochondrial genes force more activity of nuclear encoded ETC complex II to compensate the deficit of other ETC complexes encoded by mitochondrial genes, it is surprising to see less efficient conversion of fumarate from succinate, suggesting a lower activity of succinate dehydrogenase. We confirmed that the activities of these enzymes which are the ratio of substrate to product are lower in cells depleted of CAPER (S4 Fig).

Consistent with the fact that these genes are targets of c-Myc, we found changes in expressions of corresponding enzymes mediating the reactions of metabolites in glycolysis and the TCA cycle. These include lower expressions of Hexokinases (Hk), phosphoglycerate kinase (Pgk1: Pyruvate/PG), Citrate Synthase (Cs: Citrate/OAA), succinate dehydrogenase (Sdh: fumarate/succinate)s (Fig 5F). Our ChIP analyses indeed confirmed the presence of CAPER on Myc binding sites at the promoter of the Hk gene which is one of the representative targets of Myc (Fig 5F (ii)).

To further substantiate the functional relevance of these reduced key enzymes, we measured levels of NADH which are a product of the TCA cycle. We detected a dramatically lower NADH level with a slight increase in the NAD amount in cells depleted of CAPER. Addition of the NAD precursor, nicotinamide (NAM), to cells depleted of CAPER increases NADH less than controls, even though the NAM-dependent increase in NAD is comparable in both cells. (Fig 5G). This suggests that CAPER deficiency inhibits the reactions converting to NADH (but not NAD synthesis per se from NAM) even in the presence of comparable levels of NAD. Our demonstration of CAPER dependent glucose-induced NADH production, but not NAM-

dependent NADH generation, corroborates that CAPER accelerates synchronized glucose-derived carbon flux into glycolysis through the TCA cycle to produce NADH for ETCs. An end result of CAPER deficiency is a progressive deprivation of cellular ATP as shown in Fig 5H. In summary, the broad impact of CAPER on glycolysis, TCA cycle, PPP, together with direct control of the mitochondrial transcription machineries again confirms a crucial role for CAPER in cellular energy homeostasis.

CAPER is important to maintain adult life span and reproduction in *C. elegans*

To extend the conserved roles for CAPER to survival of a whole organism, we took advantage of *C. elegans* which is a powerful animal model to investigate long-term phenotypes such as life span. To examine the phenotypic outcome in a CAPER-depleted animal, we knocked down *Y55F3AM.3*, the worm ortholog of CAPER, which contains a 51% identical amino acid sequence with one SR domain and three conserved RNA binding motifs as compared to human CAPER protein (S1 Table and S6A Fig). RNA interference (RNAi) feeding of *Y55F3AM.3* starting at the first larval (L1) stages in *rrf-3 (pk1426)*, an RNAi hypersensitive strain, did not result in an overall developmental or growth defect; yet a shortened adult life span to 33% (Fig 6A (i) and S5 Table) was observed. RNAi feeding of *Y55F3AM.3* starting from the last larval (L4) stage which bypasses any development-associated effect on the adult life span further validates that RNAi inactivation of *Y55F3AM.3* in *rrf-3 (pk1426)* significantly shortens adult life span to 47% of control animals (Fig 6A (ii) and S5 Table). In addition, RNA interference of *Y55F3AM.3* starting from the first larval (L1) stages in *rrf-3 (pk1426)*, also significantly reduced brood size by 6-fold (Fig 6B) suggesting a crucial role for worm CAPER in reproductive capacity.

Notably, RNAi inactivation of *Y55F3AM.3*in transgenic animals expressing *mCherry* proteins fused with Lgg-1 which is the worm ortholog of the mammalian LC3 significantly increased the accumulation of aggregated Lgg-1 (Figs 6C and S7A). These data suggest that RNAi inactivation of *Y55F3AM.3* in parental animals induced autophagy. Like mammalian cell counterparts, RNAi inactivation of *Y55F3AM.3* in *rrf-3 (pk1426)* induced stronger signals of both lysotracker (S7B Fig) and lysosensor (S7C Fig) in multiple tissues including intestine and body wall muscles which are major metabolic organs indicating the increased lysosomal activities in RNAi inactivated day 5-old animals. These worm phenotypes strikingly support conservation of metabolic roles for CAPER in worms.

To validate the conservation of the causal bioenergetic roles for worm CAPER, we examined initial phenotypes from young animals. *Y55F3AM.3* inactivation reduces the oxygen consumption rate per animal at day 1 adulthood (Fig 6D (i)) followed by decreased ATP content from day 2 and 3 adulthood (Fig 6D (ii)); this conservation confirms that *Y55F3AM.3* inactivation causes mitochondrial dysfunction followed by energy depletion in *C. elegans*. In addition, the *Y55F3AM.3* inactivated animals did not exhibit a FCCP-dependent increase in respiration (Fig 6D (i)), indicating an impaired capacity for enhancement of respiration to combat mitochondrial stress; this result also recapitulates bioenergetic phenotype observed in mammalian cells. This deficiency likely is associated with increased oxidative stress, which is evident by a reduced GSH/GSSG ratio in the *Y55F3AM.3* inactivated animals (Fig 6D (iii)).

To further support the conservation of the transcriptional roles of CAPER, we found that mammalian and *C. elegans* CAPERs share common target genes encoding mitochondrial transcription machineries. As shown in Fig 6E, worm orthologs of mitochondrial transcription machineries that we examined are dramatically downregulated in the *Y55F3AM.3* inactivated adult worms (Fig 6E) containing decreased full length *Y55F3AM.3* (S6B Fig)

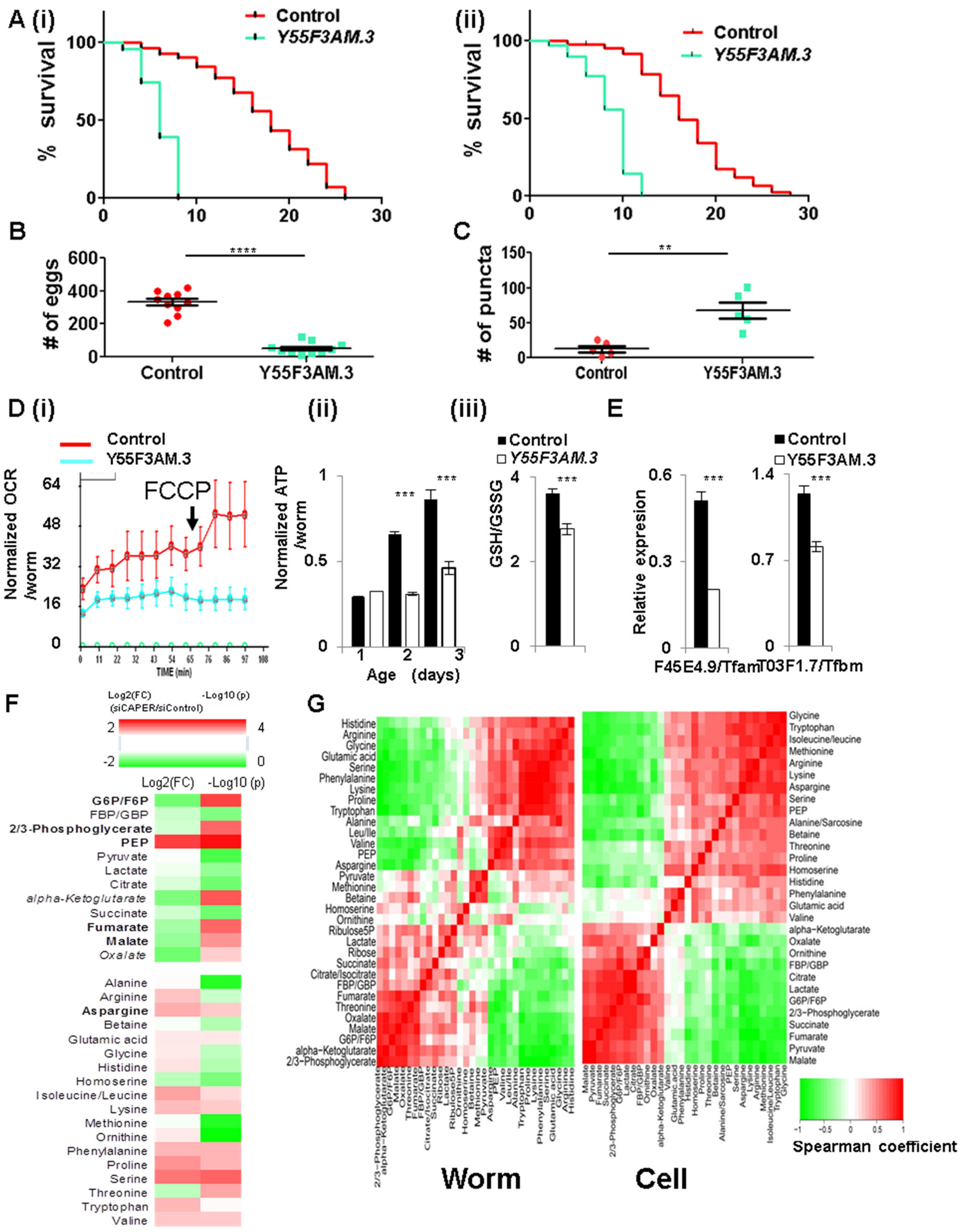
A (i)
(ii)
Control
Y55F3AM.3
% survival
B
# of eggs
****
Control
Y55F3AM.3
C
# of puncta
**
D (i)
(ii)
(iii)
E
Normalized OCR /worm
FCCP
TIME (min)
Normalized ATP /worm
***
Age (days)
GSH/GSSG
Relative expresion
F45E4.9/Tfam
T03F1.7/Tfbm
F
Log2(FC) (siCAPER/siControl)
-Log10 (p)
Log2(FC)
-Log10 (p)
G6P/F6P
FBP/GBP
2/3-Phosphoglycerate
PEP
Pyruvate
Lactate
Citrate
alpha-Ketoglutarate
Succinate
Fumarate
Malate
Oxalate
Alanine
Arginine
Aspargine
Betaine
Glutamic acid
Glycine
Histidine
Homoserine
Isoleucine/Leucine
Lysine
Methionine
Ornithine
Phenylalanine
Proline
Serine
Threonine
Tryptophan
Valine
G
Worm
Cell
Spearman coefficient

Fig 6. The conserved bioenergetic role of CAPER is essential for organismic survival and reproductive capacity in *C. elegans*. A. Graphs showing that knockdown of *Y55F3AM.3* (worm CAPER) (blue) starting from L1 stage (i) and L4 stage (ii) of rrf-3 (*pk1426*) strains reduces life span by 64% of control (red) with mean life span of 6 days and by 53% of control (red) with mean life span of 8 days, respectively. B. A graph showing the brood size from the rrf-3 (*pk1426*) strains fed with bacteria expressing either vector control (red) or the RNAi clone targeting *Y55F3AM.3 (blue)* since L1 stage. C. A quantitative graph showing a number of puncta of either control (red) or *Y55F3AM.3*-inactivated (blue) 5-day old adult Lgg-1::mCherry strain fed RNAi since L1 stage. D. Graphs showing that knockdown of *Y55F3AM.3* decreases (i) FCCP-induced OCR (n = 100) of 1-day-old adults, (ii) ATP content per worm from 2-day-old and 3-day-old but not 1-day-old adult animals (n = 30 for each set) of *rrf-3*(*pk1426*) strains fed with the indicated RNAi clones since L1 stage. (iii) A bar graph showing the GSH/GSSG from 2-day-old worms of *rrf-3*(*pk1426*) strains fed with the indicated RNAi clones since L1 stage. E. Graphs showing the results of qRT-PCR of that knockdown of *Y55F3AM.3* reduce representative worm target genes from 2-day-old adult animals. Mean value with standard of error of means are presented. F. RNA inactivation of *Y55F3AM.3* disturbs carbon and nitrogen balance in whole worms. A heat map showing altered metabolites by Log_2 (FC: fold change) (left two colums-24 hour and 48 hour samples) and by Log_{10}(p values) (p values adjusted for multiple comparisons using Benjamini-Hochberg method) (right two columns). Scale of red (positive values) and green (negative values) are represented as a color code on top of the figure. G. Conservation of CAPER-dependent metabolic programs. Comparison of CAPER-dependent association among metabolites were calculated by Fisher's z transformation using Spearman correlation coefficients 91% of pairs of each metabolites are interchangeable.

doi:10.1371/journal.pgen.1005116.g006

To test conservation of the CAPER-dependent metabolite signature, we measured relative quantities of worm metabolites relevant to bioenergetic pathways including glycolysis, TCA cycle and amino acids—all of which are shown to be altered in mammalian cells. The majority of examined worm metabolites were altered by *Y55F3AM.3* inactivation in a very similar manner that was observed in mammalian cells (Fig 6F).

To further substantiate the conservation in CAPER-mediated metabolic programs between worm and mammalian cells, we compared the correlation distance between pairs of worm metabolites altered by *Y55F3AM.3* inactivation to the correlation between mammalian counterparts by calculating Fisher's z distribution of Spearman correlation coefficients in worms and cells (S6 Table). Strikingly, Fisher's z distribution revealed that only 9% of metabolic associations are statistically different; the results suggest that 91% of correlations among metabolites are conserved in worms and mammalian counterparts (Fig 6G and S6 Table). Overall, our experimental data indicate that the crucial metabolic roles assigned to CAPER are evolutionarily conserved and that CAPER is a master nodal regulator dedicated to maintain organism survival and reproduction.

Discussion

CAPER is a vital metabolic accelerator that promotes growth and reproduction and maintains life-span for species survival

Reproductive capacity and life span are the most influential determinants for evolutionary prevalence of a species. Multiple nutrients and metabolic reactions must exist to generate energy and to synthesize the macromolecular constituents required for reproductive span and capacity [20–25]. Nevertheless, the conserved mechanisms that eukaryotes share to propagate and integrate nutrient signaling to modulate growth, reproduction and life span are not completely defined.

Our study presents evidence that CAPER, which is highly conserved among eukaryotes, represents a unifying master regulator node that synchronizes multiple pathways involved in nutrient utilization by controlling other downstream nodal regulators that regulate expression of key metabolic enzymes for enhancement of growth, reproduction and life span. CAPER is a vital energy generator for both glycolysis and mitochondrial function. Many prior studies have detailed the absolute inverse relationships between glycolysis and mitochondrial respiration, exemplified by the Warburg effect. However, our studies substantiate that glycolysis is, in fact, important to provide carbon flux for the TCA cycle and for a mitochondrial proton gradient for enhancement of mitochondrial respiratory capacity. In addition to glycolysis, CAPER regulates various anaplerotic carbon fluxes from fatty acid beta oxidation and amino acid

catabolism to replenish the TCA cycle. Importantly, these metabolic events clearly occur prior to autophagy or growth/survival phenotypes in both mammalian cells and *C. elegans* that are deprived of CAPER, supporting our conclusion that these initial metabolic defects are causal factors for induction of the later associated phenotypes.

CAPER maintains intrinsic mitochondrial ETC activities by direct transcriptional control of mitochondrial transcriptional machinery genes. Given the critical requirement for ATP for the anabolic syntheses of macromolecules, CAPER-dependent coordination of the energy supply and macromolecular synthesis for increased metabolic demand and nutrient status provides a means to optimize levels of ATP for growth and survival.

CAPER maintains a cellular balance between metabolites containing carbon and those that contain nitrogen. During preparation of our manuscript, balanced ratio of macronutrients, particularly protein restriction not the caloric intake per se has been reported to be more important in longevity [2,26]. Our study not only supports this notion but also provides the molecular basis how eukaryotes maintain metabolic balance between carbon-nitrogen metabolites via CAPER. Multiple metabolic processes that utilize carbon sources include those of glucose, fatty acids and amino acids; their homeostatic dependence on CAPER suggests an additional essential role for it in cellular carbon flux to modulate cellular nitrogen metabolites by linking glycolysis and protein catabolism such as autophagy, as well as the TCA cycle. Given the impressive number of metabolic pathways that we found to be harmonized by CAPER, our study suggests CAPER as a major rheostat that enhances the flow of carbon and nitrogen elements into the TCA cycle to support energy generation and cellular metabolic balance.

CAPER safeguards cellular redox homeostasis and mediates an intimate link between GSH and glucose metabolism. Considering the well-known adverse effects of glucose metabolism and the strong beneficial impact of caloric restriction (CR) on various health issues including life span [27], it was unexpected to see CAPER mimic the beneficial actions of CR to extend life span, yet still boost nutrient metabolism. Nevertheless, it is logical that cells devise mechanisms/molecules by which they can be protected from oxidative stresses while they enhance metabolic flux. By integrating energy metabolism, anabolic biosynthesis and antioxidant defense to increases growth, reproductive capacity, motility and ultimately life span, CAPER functions as such a molecule.

CAPER is a co-evolutionary node that propagates nutrient signaling by integrating nutrient-dependent mitochondrial function and stress responses and coordinating nuclear-mitochondrial transcriptional responses

Optimal execution of nutrient metabolism is an essential prerequisite for species survival in response to changes in the environment and food availability. Along these lines, a variety of studies have identified various transcription factors as sensors of glucose such as LXR which can directly bind glucose [28], FXR [29] or c-MYC [30] and Chrebp [31].

Because the mitochondrial genome encodes components of the electron transport chain and mitochondrial translation system that are essential for mitochondrial functions, eukaryotes must coordinate these two genomes for maximal metabolic performance. This bigenomic system in eukaryotes demands that co-regulatory nodes be evolved to carry out such orchestration. In fact, such a node(s) likely would be under constant selective pressure so as to be responsive to changing nutritional environmental adaptations. We propose that our observed CAPER-mediated regulation of both nuclear and mitochondrial transcriptional programs provides such a means to coordinate energy and macromolecule synthesis through glycolysis, mitochondrial oxidative phosphorylation, fatty acid oxidation and amino acid metabolism,

thereby providing a strong metabolic advantage to the organism during evolutionary selection. CAPER senses quantitative nutritional changes by changing its own quantity and functions as a master hub to conduct direct transcriptional regulation of other nodal regulators that include c-Myc and Gabpa (Fig 7).

CAPER's unique properties are substantiated further by our data demonstrating that different phenotypes are produced by its knockdown, as compared to those of PGC-1α or SRC-2 coactivators. In particular, PGC-1α also regulates a mitochondrial transcription program [10], but unlike CAPER, PGC-1α regulates the number of mitochondria by inducing mitochondrial biogenesis. Also, CAPER is induced when high levels of nutrients are present, in contrast to PGC-1α which is induced upon fasting [32]. These results clearly distinguish their respective roles in nutrient metabolism and mitochondrial function.

Given the significant metabolic roles for CAPER for achieving optimal nutrient utilization and for providing stress-resistance including bioenergetic restriction for maximal nutrient dependent cellular growth, it could be expected that cancers would prefer higher levels of CAPER. Supporting this notion, the CAPER gene was found to be amplified in lung and breast cancers (Tumorscape from Broad institute: www.broadinstitute.org/broadinstitute) and various GI cancers [33]. Levels of CAPER transcripts also are higher in brain cancer (oncomine.org), ovarian cancer [34], and metastatic prostate cancer (GEO profile) and it has been found to contribute to carcinogenesis in mouse models of breast [5] and prostate metastases [35].

Materials and Methods

Reagents, cell culture, transient transfection, and determination of cell proliferation

Detailed information is provided in the Extended methods.

ATP measurement

Cellular ATP was measured by ATP lite kit (Perkin Elmer) according to manufacturer's instruction. After transfecting siRNA, 2000 cells were seeded and grown onto 96 well plates. After 24, 48 and 72 hours, cells were lysed with lysis buffer and we measured cellular ATP levels using luminometer.

Immunofluorescence

Transfected AML12 cells were fixed, permeabilized, and stained with the indicated markers as described previously [36]. Deconvolution microscopy was performed with a Zeiss Axiovert S100 TV microscope and a DeltaVision Restoration Microscopy System (Applied Precision, Inc.). Z series focal planes were digitally imaged and deconvolved with the DeltaVision constrained iterative algorithm. All image files were digitally processed for presentation with Adobe Photoshop.

Mitochondrial analyses

Mitochondrial numbers were measured by both using qPCR to measure the ratio between mtDNA (mtND2) and nuclear DNA (cyclophilin) and staining with Mitotracker Red CMXRos (Molecular Probe). The mitochondrial oxygen consumption rate (OCR) and extracellular acidification rate (ECAR) were examined by XF24 analyzer (Seahorse bioscience) according to the manufacturer's instructions. Briefly, cells were transfected with siRNAs and 3000 cells were seeded onto XF24 plates. After 24 hours, cells were subjected to the XF24 analyzer upon instructions.

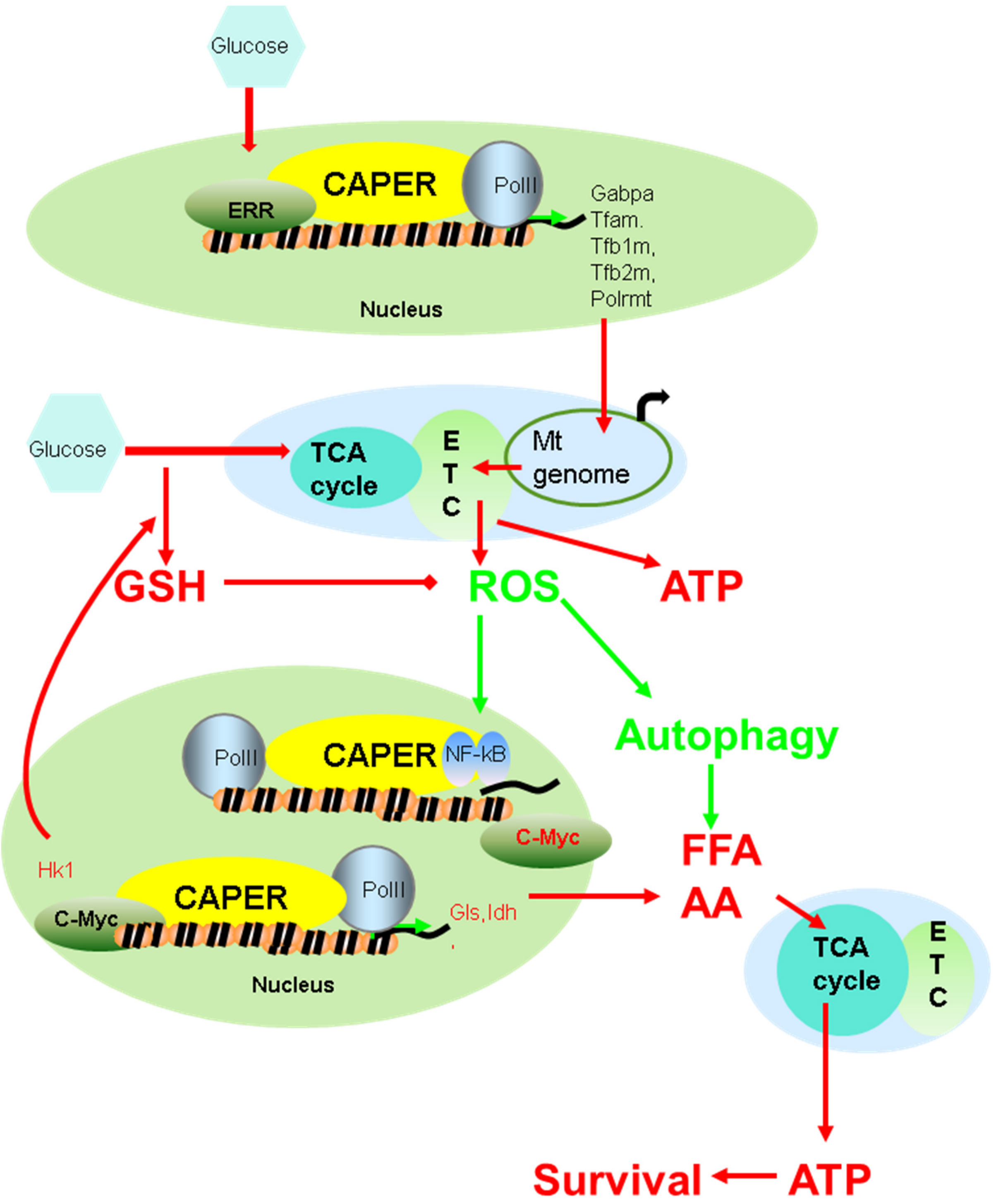

Fig 7. A schematic model suggesting how CAPER functions.

doi:10.1371/journal.pgen.1005116.g007

Reverse transcription and quantitative real-time PCR

QRT-PCR analyses of endogenous mRNAs were performed. Total RNA was isolated from 12-well culture dishes using the RNeasy kit (QIAGEN) followed by first strand cDNA synthesis

using Superscript II (Invitrogen) according to the manufacturer's instruction. Real-time PCR reactions were performed using the AIB Step One Plus sequence detection system (Applied Biosystems) with SyBr Green reaction mixture according to the manufacturer's instruction. Means and S.E.M from the three or five independent replicates were then calculated. The Student's t test was used to evaluate the data. To avoid variations from different samples, the relative mRNA level of each gene was normalized against the beta-2-microgloblulin mRNA content of the same sample unless stated otherwise. Primer sequences are available upon request.

Western blot analyses and quantitation

Whole cell lysates were prepared as described previously [37]. To maintain the integrity and phosphorylation status of proteins, proteinase inhibitors and phosphatase inhibitors (Gene Depot) were added freshly. Western blot analyses were performed following standard procedures using Immune star (Bio Rad). Antibodies were from various sources: Anti-tubulin (Sigma), anti-NF-κB and anti-CAPER antibody (Bethyl) antibody is from Novus. Each band was subjected to quantitation as follows: relative fold-changes were presented by setting up each normalized protein to "1"- relative to the amount shown in lane 1 unless stated otherwise. Quantification of the relative intensity from the image was done using Image J free software (NIH, USA) [38].

Immunoprecipitation and chromatin immunoprecipitation

Immunoprecipitation and GST pull down assays were essentially performed as previously described [37]. To avoid masking signals by IgG, we used True blot IP beads (eBioscience). ChIP assay was performed using the Simple ChIP enzymatic Chromatin IP kit (Cell Signaling) according to manufacturer's instructions. The Epitect ChIP qPCR primers for NF-κB binding sites for c-Myc and Frap1 were purchased from Qiagen.

ATP measurement

Cellular ATP was measured with the ATP Lite kit (Perkin Elmer) according to manufacturer's instructions.

Metabolomic profiling and metabolite flux analyses

Metabolomic profiling is essentially performed as previously reported [19] and detailed procedures are provided in the extended methods (S1 Text). For metabolite flux analyses, AML12 cells are transfected with either siControl or siCAPER. After 12 hours, we changed growth media with glucose starvation media (DMEM with no glucose supplemented with insulin-transferrin-selenium). We changed media with DMEM containing 25mM [U-^{13}C6]-D-glucose 3 hours (3h) or 5 min (0h) prior to harvest. Cells are washed with PBS and added Methanol-water followed by snap frozen in liquid nitrogen. Samples were analyzed by analyses.

Repetitions of experiments

Experiments in all figures were repeated at least three times except metabolomics and RNA-seq which are as noted in the text.

Statistical analyses

The difference between siControl to siCAPER were statistically analyzed by student's t-test using GraphPad software ($p<0.05$ (*), $p<0.01$ (**) and $p<0.005$ (***)) unless stated differently in the legend.

Supporting Information

S1 Fig. Specificity of CAPER dependent phenotype. A. Knockdown of CAPER suppresses cell proliferation starting from 3 days. (i) A growth curve showing overall growth kinetics. Cell numbers were counted at the indicated days after transfecting either siControl (black solid line) or siCAPER (black dotted line). A graph presents average and SEM of the triplicate as an error bar. Statistical analysis by student's t-test was performed (** $p<0.01$). (ii) A representative microscopic picture showing the result of crystal violet staining 5 days after transfection with indicated siRNAs. B. The efficacy and duration of siRNA targeting CAPER was confirmed by Western blots (i) and qRT-PCR analyses (ii). Amount of CAPER protein and mRNA were shown at the indicated days after transfecting each siRNA (-: control siRNA, +: siRNA targeting CAPER). C. A representative microscopic picture (left panel) and a quantitative graph (right panel) showing the result of crystal violet staining 6 days after transfection with indicated siRNAs targeting CAPER or control. Bar graphs present the mean value of three replicates and error bars represent SEM. D. A representative microscopic picture (left panel) and a quantitative graph (right panel) showing the result of crystal violet staining 6 days after transfection with indicated siRNAs targeting CAPER or control followed by transfection of either hrGFP or CAPER mRNAs. Bar graphs present the mean value of three replicates and error bars represent SEM. E. Fluorescence pictures (upper panel) and the corresponding quantitative graphs (lower panel) showing the relative abundance of mouse Lamp1(left panel) and Lamp2 (right panel) from the cells after transfection with either siControl (left panel) or siCAPER. Pictures were taken at 48 hours after siRNA transfection as described in the experimental procedure (right panel). A scale bar is present. Staining intensity was quantified by Image J software (NIH) and statistical significance was calculated by student's t-test using GraphPad software.
(TIF)

S2 Fig. Uniqueness of CAPER dependent phenotype. A. Microscopic pictures showing morphology of cells at 2 days after transfecting with siControl, siCAPER and siPGC-1α. B. Crystal violet staining showing the effect of siControl and siPGC-1α in combination with the indicated glucose concentration (mM) on cell proliferation. Photos were taken 6 days after siRNA transfection. C. A graph showing the result from qRT-PCR to measure mRNA levels of PGC-1α (i) and Tfb1m (ii) to confirm the efficacy of siPGC-1α(i) and its effect on glucose dependent induction of Tfb1m (ii), respectively. Levels of each transcript are normalized with the amount of β-2MG as an internal control. Each bar represents the mean value of normalized mRNA levels of each gene from triplicates with an error bar (SEM). Data were analyzed by t-test using GraphPad software. D. (i) Crystal violet staining showing the effect of siControl, siCAPER and siSRC2 on cell proliferation. Photos were taken 6 days after siRNA transfection. (ii) A graph showing the qRT-PCR result measuring mRNA of SRC2 after transfecting with the indicated siRNAs. Quantitation and statistical analyses are as described in the Materials and Methods. E. Microscopic pictures showing morphology of the cells transfected with either the indicated siRNAs only or in combination of Adenovirus encoding vector or SRC-2. 6 hours after siRNA, adenoviruses were infected with 8microgram/ml of polybrene in serum free media for overnight. Photos were taken 3 days after adenovirus infection. F. A representative microscopic picture shows the morphology of primary hepatocytes 2 days after transfection with indicated siRNAs

targeting CAPER or control. G. Representative microscopic pictures show the morphology of Hepa1-6 cells 2 days after transfection (upper panel) and the result of crystal violet staining 6 days after transfection (lower panel) with indicated siRNAs targeting CAPER or control.
(TIF)

S3 Fig. Cell cycle and apoptosis in cells deficient CAPER. A. FACS analyses showing proportion of Annexin V positive cells from cells depleted CAPER (siCAPER) and control (siControl). Annexin V staining was performed 3 days after transfecting either control (upper panel) or siCAPER (lower panel). B. Histogram showing the FACS analyses of PI staining at 36 hours (left) and 72 hours (right) after transfecting either control (upper panel) or siCAPER (lower panel). C. QRT-PCR showing the kinetic expression of p53, p21, p16/Ink4 and Nfe2l2/Nrf2 transcripts at the indicated times after transfection. Quantitation and statistical analyses were carried out as described in S2C Fig.
(TIF)

S4 Fig. Fold change of kinetic metabolic rates in cells deficient CAPER to one in control. A graph showing the computed ratio of the rate of average total quantities of two C13 labeled metabolites in siCAPER to ones in siControl cells. We computed the ratio of two indicated metabolites from cells treated with siCAPER or siControl. We then calculate the mean value of these ratios of metabolites in three biological replicates. Using these mean values of metabolic rate, we computed the ratio of these values of cells treated with siCAPER to ones with siControl. Each replicate is a pool of biological duplicate.
(TIF)

S5 Fig. Treatment of NAC and F1, 6-BP is sufficient to suppress autophagy-mediated vacuoles but not cell proliferation. A. Microscopic pictures showing the result of crystal violet staining (left) and morphology (right) of the cells treated with two different siRNAs targeting CAPER (siCAPER1 and siCAPER 2) followed by treating NAC and F1,6-BP. B. Quantitative graphs of qRT-PCR showing the negligible effect of NAC treatment on the levels of c-Myc, Tfam, Tfb1m and Tfb2m from the cells treated with either control or siCAPER. C. Quantitative graphs of qRT-PCR showing the comparable ratio of mitochondrial DNA copy number to nuclear DNA copy number with or without NAC.
(TIF)

S6 Fig. Conservation between *Y55F3AM.3* and hRBM39. A. Amino acid sequence alignment of *Y55F3AM.3* and hRBM39. B. A qRT-PCR showing the reduced full-length *Y55F3AM.3* in both 1-day (left) and 3-day (right) old worms fed bacteria expressing either control (balck) or *Y55F3AM.3* (white) RNAi clones.
(TIF)

S7 Fig. RNAi inactivation of *Y55F3AM.3* induces autophagy in worms. A. Representative microscopic pictures showing the signals of mCherry::Lgg-1 in 5-day old adult animals fed bacteria expressing either control or *Y55F3AM.3* RNAi clones since L1 stage. B. Representative microscopic pictures show the lysotracker staining of either control or *Y55F3AM.3*-inactivated 3 (i) or 5-day (ii) old adult rrf-3 (*pk1426*) strains fed with the indicated RNAi clones since L1 stage. C. Representative microscopic pictures show the lysosensor DND-189 staining of either control or *Y55F3AM.3*-inactivated 5-day old adult rrf-3 (*pk1426*) strains fed with the indicated RNAi clones since L1 stage.
(TIF)

S1 Table. Pairwise alignment scores to compare human RBM39 gene with orthologs from 20 representative species.
(DOCX)

S2 Table. eQTL located in human RBM39 gene.
(DOCX)

S3 Table. Pathway analyses of RNA-seq dataset.
(XLSX)

S4 Table. Metabolomic data.
(XLSX)

S5 Table. Life span analysis.
(XLSX)

S6 Table. Comparison of Spearman's correlation matrices from cells and worms.
(XLSX)

S1 Text. Extended methods.
(DOCX)

Acknowledgments

We thank Drs. Bruce Spiegelman and Donald McDonnell for Gabpa-luc and ERR α, respectively. We are thankful Dr. Charles Foulds and Ross Hamilton for technical help with nuclear extracts, Vasantha Putluri for assistance with flux analyses. Mitochondrial assays using a XF 24 analyzer were carried out at our metabolic core at DERC with the help of Dr. Pradip Saha. Viral work was supported by the Diabetes and Endocrinology Research Center at Baylor College of Medicine with the help of Dr. Kazuhiro Oka. We acknowledge the support of the "Genomic and RNA Profiling (GARP)" Core at BCM and Dr. Lisa D. White and "the Cytometry and Cell Sorting Core at BCM and the assistance of Amanda N. White. Microscopic analyses were carried out in the Integrated Microscopic Core at Baylor College of Medicine.

Author Contributions

Conceived and designed the experiments: YKK BWO. Performed the experiments: YKK OI NP. Analyzed the data: SM GM AS AT QM. Contributed reagents/materials/analysis tools: MW. Wrote the paper: YKK BWO. Mass spectrometry analysis of metabolomics: CBN AS. Discussion about the results: MW MJT SYT. Review of manuscript: DL MJT SYT.

References

1. Gomes AP, Price NL, Ling AJ, Moslehi JJ, Montgomery MK, et al. (2013) Declining NAD(+) induces a pseudohypoxic state disrupting nuclear-mitochondrial communication during aging. Cell 155: 1624–1638. doi: 10.1016/j.cell.2013.11.037 PMID: 24360282
2. Solon-Biet SM, McMahon AC, Ballard JW, Ruohonen K, Wu LE, et al. (2014) The ratio of macronutrients, not caloric intake, dictates cardiometabolic health, aging, and longevity in ad libitum-fed mice. Cell Metab 19: 418–430. doi: 10.1016/j.cmet.2014.02.009 PMID: 24606899
3. Jung DJ, Na SY, Na DS, Lee JW (2002) Molecular cloning and characterization of CAPER, a novel coactivator of activating protein-1 and estrogen receptors. J Biol Chem 277: 1229–1234. PMID: 11704680
4. Dowhan DH, Hong EP, Auboeuf D, Dennis AP, Wilson MM, et al. (2005) Steroid hormone receptor coactivation and alternative RNA splicing by U2AF65-related proteins CAPERalpha and CAPERbeta. Mol Cell 17: 429–439. PMID: 15694343

5. Mercier I, Casimiro MC, Zhou J, Wang C, Plymire C, et al. (2009) Genetic Ablation of Caveolin-1 Drives Estrogen-Hypersensitivity and the Development of DCIS-Like Mammary Lesions. The American Journal of Pathology 174: 1172–1190. doi: 10.2353/ajpath.2009.080882 PMID: 19342371
6. Dutta J, Fan G, Gélinas C (2008) CAPERα Is a Novel Rel-TAD-Interacting Factor That Inhibits Lymphocyte Transformation by the Potent Rel/NF-κB Oncoprotein v-Rel. Journal of Virology 82: 10792–10802. doi: 10.1128/JVI.00903-08 PMID: 18753212
7. Wu JC, Merlino G, Fausto N (1994) Establishment and characterization of differentiated, nontransformed hepatocyte cell lines derived from mice transgenic for transforming growth factor alpha. Proceedings of the National Academy of Sciences 91: 674–678. PMID: 7904757
8. Zhou J, Tan S-H, Nicolas V, Bauvy C, Yang N-D, et al. (2013) Activation of lysosomal function in the course of autophagy via mTORC1 suppression and autophagosome-lysosome fusion. Cell Res 23: 508–523. doi: 10.1038/cr.2013.11 PMID: 23337583
9. Klionsky DJ, Abdalla FC, Abeliovich H, Abraham RT, Acevedo-Arozena A, et al. (2012) Guidelines for the use and interpretation of assays for monitoring autophagy. Autophagy 8: 445–544. PMID: 22966490
10. Finck BN, Kelly DP (2006) PGC-1 coactivators: inducible regulators of energy metabolism in health and disease. J Clin Invest 116: 615–622. PMID: 16511594
11. Chopra AR, Kommagani R, Saha P, Louet JF, Salazar C, et al. Cellular energy depletion resets whole-body energy by promoting coactivator-mediated dietary fuel absorption. Cell Metab 13: 35–43. doi: 10.1016/j.cmet.2010.12.001 PMID: 21195347
12. Bensaad K, Vousden KH (2007) p53: new roles in metabolism. Trends in Cell Biology 17: 286–291. PMID: 17481900
13. Villeneuve NF, Sun Z, Chen W, Zhang DD (2009) Nrf2 and p21 regulate the fine balance between life and death by controlling ROS levels. Cell Cycle 8: 3255–3256. PMID: 19806015
14. Lee J, Giordano S, Zhang J (2012) Autophagy, mitochondria and oxidative stress: cross-talk and redox signalling. Biochem J 441: 523–540. doi: 10.1042/BJ20111451 PMID: 22187934
15. Owusu-Ansah E, Yavari A, Mandal S, Banerjee U (2008) Distinct mitochondrial retrograde signals control the G1-S cell cycle checkpoint. Nat Genet 40: 356–361. doi: 10.1038/ng.2007.50 PMID: 18246068
16. Scarpulla RC (2008) Nuclear control of respiratory chain expression by nuclear respiratory factors and PGC-1-related coactivator. Ann N Y Acad Sci 1147: 321–334. doi: 10.1196/annals.1427.006 PMID: 19076454
17. Jazwinski SM (2013) The retrograde response: when mitochondrial quality control is not enough. Biochim Biophys Acta 1833: 400–409. doi: 10.1016/j.bbamcr.2012.02.010 PMID: 22374136
18. Sreekumar A, Poisson LM, Rajendiran TM, Khan AP, Cao Q, et al. (2009) Metabolomic profiles delineate potential role for sarcosine in prostate cancer progression. Nature 457: 910–914. doi: 10.1038/nature07762 PMID: 19212411
19. Putluri N, Shojaie A, Vasu VT, Vareed SK, Nalluri S, et al. (2011) Metabolomic profiling reveals potential markers and bioprocesses altered in bladder cancer progression. Cancer Res 71: 7376–7386. doi: 10.1158/0008-5472.CAN-11-1154 PMID: 21990318
20. Valcourt JR, Lemons JM, Haley EM, Kojima M, Demuren OO, et al. (2012) Staying alive: metabolic adaptations to quiescence. Cell Cycle 11: 1680–1696. doi: 10.4161/cc.19879 PMID: 22510571
21. Harnisch JM, Harnisch PH, Harnisch DR Sr (2012) Family Medicine Obstetrics: Pregnancy and Nutrition. Primary Care: Clinics in Office Practice 39: 39–54. doi: 10.1016/j.pop.2011.11.003 PMID: 22309580
22. Della Torre S, Rando G, Meda C, Stell A, Chambon P, et al. Amino Acid-Dependent Activation of Liver Estrogen Receptor Alpha Integrates Metabolic and Reproductive Functions via IGF-1. Cell Metabolism 13: 205–214. doi: 10.1016/j.cmet.2011.01.002 PMID: 21284987
23. Michalakis K, Mintziori G, Kaprara A, Tarlatzis BC, Goulis DG (2013) The complex interaction between obesity, metabolic syndrome and reproductive axis: A narrative review. Metabolism.
24. Della Torre S, Rando G, Meda C, Stell A, Chambon P, et al. (2011) Amino acid-dependent activation of liver estrogen receptor alpha integrates metabolic and reproductive functions via IGF-1. Cell Metab 13: 205–214. doi: 10.1016/j.cmet.2011.01.002 PMID: 21284987
25. Klosinska MM, Crutchfield CA, Bradley PH, Rabinowitz JD, Broach JR (2011) Yeast cells can access distinct quiescent states. Genes Dev 25: 336–349. doi: 10.1101/gad.2011311 PMID: 21289062
26. Levine ME, Suarez JA, Brandhorst S, Balasubramanian P, Cheng CW, et al. (2014) Low protein intake is associated with a major reduction in IGF-1, cancer, and overall mortality in the 65 and younger but not older population. Cell Metab 19: 407–417. doi: 10.1016/j.cmet.2014.02.006 PMID: 24606898

27. Colman RJ, Anderson RM, Johnson SC, Kastman EK, Kosmatka KJ, et al. (2009) Caloric Restriction Delays Disease Onset and Mortality in Rhesus Monkeys. Science 325: 201–204. doi: 10.1126/science.1173635 PMID: 19590001
28. Mitro N, Mak PA, Vargas L, Godio C, Hampton E, et al. (2007) The nuclear receptor LXR is a glucose sensor. Nature 445: 219–223. PMID: 17187055
29. Duran-Sandoval D, Mautino G, Martin G, Percevault F, Barbier O, et al. (2004) Glucose regulates the expression of the farnesoid X receptor in liver. Diabetes 53: 890–898. PMID: 15047603
30. Briata P, Laurino C, Gherzi R (1989) c-myc Gene expression in human cells is controlled by glucose. Biochemical and Biophysical Research Communications 165: 1123–1129. PMID: 2558650
31. Li MV, Chen W, Harmancey RN, Nuotio-Antar AM, Imamura M, et al. (2010) Glucose-6-phosphate mediates activation of the carbohydrate responsive binding protein (ChREBP). Biochem Biophys Res Commun 395: 395–400. doi: 10.1016/j.bbrc.2010.04.028 PMID: 20382127
32. Yoon JC, Puigserver P, Chen G, Donovan J, Wu Z, et al. (2001) Control of hepatic gluconeogenesis through the transcriptional coactivator PGC-1. Nature 413: 131–138. PMID: 11557972
33. Sillars-Hardebol AH, Carvalho B, Belien JA, de Wit M, Delis-van Diemen PM, et al. (2012) CSE1L, DIDO1 and RBM39 in colorectal adenoma to carcinoma progression. Cell Oncol (Dordr) 35: 293–300. doi: 10.1007/s13402-012-0088-2 PMID: 22711543
34. Ramakrishna M, Williams LH, Boyle SE, Bearfoot JL, Sridhar A, et al. (2010) Identification of candidate growth promoting genes in ovarian cancer through integrated copy number and expression analysis. PLoS One 5: e9983. doi: 10.1371/journal.pone.0009983 PMID: 20386695
35. Ding Z, Wu C-J, Chu GC, Xiao Y, Ho D, et al. (2011) SMAD4-dependent barrier constrains prostate cancer growth and metastatic progression. Nature 470: 269–273. doi: 10.1038/nature09677 PMID: 21289624
36. Amazit L, Pasini L, Szafran AT, Berno V, Wu RC, et al. (2007) Regulation of SRC-3 intercompartmental dynamics by estrogen receptor and phosphorylation. Mol Cell Biol 27: 6913–6932. PMID: 17646391
37. Kang YK, Schiff R, Ko L, Wang T, Tsai SY, et al. (2008) Dual roles for coactivator activator and its counterbalancing isoform coactivator modulator in human kidney cell tumorigenesis. Cancer Res 68: 7887–7896. doi: 10.1158/0008-5472.CAN-08-1734 PMID: 18829545
38. McLean-Bennett LoA (2011) Densitometry of Western blots using Image J Software.
